# Neoadjuvant PD-1/PD-L1 inhibitors combined with chemotherapy in gastric cancer and gastroesophageal junction adenocarcinoma: a systematic review and meta-analysis of single-arm studies

**DOI:** 10.3389/fmed.2025.1625259

**Published:** 2025-08-05

**Authors:** Jun Leng, Min Liu, Qianwen Wang, Huaiwu Jiang, Jin Chen

**Affiliations:** ^1^North Sichuan Medical College, Nanchong, Sichuan, China; ^2^Department of General Surgery, Mian Yang 404 Hospital, Mianyang, Sichuan, China; ^3^Department of Gastrointestinal Surgery, Mianyang Hospital, Mianyang, Sichuan, China

**Keywords:** combination therapy, efficacy, meta-analysis, neoadjuvant immunotherapy, gastric cancer/gastroesophageal junction tumors, safety, PD-1/PD-L1 inhibitors

## Abstract

**Background:**

Neoadjuvant chemotherapy, particularly neoadjuvant immunotherapy, has achieved significant progress in treating gastric cancer (GC) and gastroesophageal junction (GEJ) adenocarcinoma. While PD-1/PD-L1 inhibitors have improved survival outcomes in some patients, the efficacy of combining neoadjuvant chemotherapy with PD-1/PD-L1 inhibitors remains insufficiently validated.

**Objective:**

This study aims to evaluate the efficacy and safety of neoadjuvant chemotherapy combined with PD-1/PD-L1 inhibitors in GC/GEJ adenocarcinoma and enhance statistical power through meta-analysis.

**Methods:**

We systematically searched PubMed, Embase, Web of Science, and the Cochrane Library up to October 5, 2024, for original clinical studies on neoadjuvant PD-1/PD-L1 inhibitors combined with chemotherapy for GC/GEJ adenocarcinoma. Eligible studies were evaluated using the Methodological Index for Non-Randomized Studies (MINORS) Criteria. Meta-analysis was performed using R 4.4.2 software.

**Results:**

A total of 12 studies involving 428 patients were included. The primary efficacy outcomes were pathological complete response (pCR) and major pathological response (MPR), while secondary outcomes included disease control rate (DCR) and the rate of lymph node downstaging to ypN0. The effect size (ES) for pCR was 20.94% [95% CI: 0.1698; 0.2518], and for MPR, the ES was 55.86% [95% CI: 0.4891; 0.6271]. Safety was evaluated using the incidence of grade ≥3 treatment-related adverse events (trAEs), immune-related adverse events (irAEs), postoperative complications, and R0 resection rate. The meta-analysis revealed that 406 patients underwent surgical intervention, with 88 achieving pCR. The pooled effect size for R0 resection rate was 95.2% [95% CI: 0.896; 0.989]. The ES values for grade ≥3 adverse events, immune-related adverse events, and postoperative complications were 0.54 [95% CI: 0.30; 0.77], 0.17 [95% CI: 0.07; 0.31], and 0.28 [95% CI: 0.15; 0.44], respectively.

**Conclusion:**

Neoadjuvant PD-1/PD-L1 inhibitor-based chemotherapy demonstrates promising therapeutic efficacy and safety in patients with gastric and gastroesophageal junction (GEJ) cancer. However, limitations such as small sample sizes and insufficient follow-up duration in current studies highlight the need for further randomized controlled trials and multicenter research to establish optimal treatment strategies.

**Systamatic review registration:**

Identifier, CRD42024619916.

## 1 Introduction

In the 21st century, gastric cancer remains a major global health threat, ranking as the fourth leading cause of cancer-related mortality (7.7%), following lung (18%), colorectal (9.4%), and liver cancers (8.3%) (1). Over the past half-century, declining *Helicobacter pylori* prevalence and improved food preservation have significantly reduced the incidence and mortality of non-cardia gastric cancer (2). However, the incidence of gastroesophageal junction (GEJ) cancer has risen in some countries. Although R0 resection rates for GEJ cancer are comparable to those for non-cardia gastric cancer, systemic recurrence rates are significantly higher (*p* = 0.031), and median survival is markedly shorter (26 months vs. 69 months; *p* < 0.001) (3).

Surgical resection remains the primary curative approach for gastric cancer and the most effective method to prolong patient survival (4). In operable gastric or lower esophageal adenocarcinoma, neoadjuvant chemotherapy reduces tumor size and stage, significantly increases R0 resection rates, and improves progression-free survival and overall survival (5). In recent years, immune checkpoint inhibitors targeting cytotoxic T-lymphocyte-associated antigen 4 (CTLA-4), programmed death (PD)-1, and PD-ligand 1 (PD-L1) have demonstrated durable responses in subsets of patients with metastatic disease (6). With the expanding use of PD-1/PD-L1 inhibitors in clinical practice (7, 8), emerging evidence suggests that combination therapies incorporating anti-PD-1 agents may represent the future of cancer immunotherapy (9, 10). This study aims to consolidate evidence for neoadjuvant PD-1 inhibitor therapy in gastric cancer and propose strategies for optimizing treatment regimens in G/GEJ tumors.

## 2 Materials and methods

This systematic review and meta-analysis were conducted and reported in accordance with the PRISMA guidelines. The study protocol was registered on PROSPERO (Registration ID: CRD42024619916).

### 2.1 Search strategy

We systematically searched health-related databases, including PubMed, Web of Science, Embase, and the Cochrane Library. Search strings incorporated Medical Subject Headings (MeSH) terms such as “gastric cancer,” “neoadjuvant therapy,” and “Esophagogastric Junction,” combined in various Boolean configurations. The search was limited to studies published from database inception to October 2024. Two researchers independently screened and categorized retrieved studies. Discrepancies were resolved through full-text review and discussion with a third reviewer. Multiple search iterations were performed to ensure comprehensiveness and accuracy.

### 2.2 Inclusion and exclusion criteria

#### 2.2.1 Inclusion criteria

1. Participants diagnosed with gastroesophageal junction (GEJ) or gastric cancer, regardless of sex, age, anatomical subsite, or tumor stage;
2. Treatment protocols involving PD-1/PD-L1 inhibitors combined with neoadjuvant therapy;
3. Reported outcomes including pathological complete response (pCR), major pathological response (MPR), objective response rate (ORR), event-free survival (EFS), overall survival (OS), and incidence of grade ≥3 treatment-related adverse events (TRAEs) or immune-related adverse events.

#### 2.2.2 Exclusion criteria

Animal studies, *in vitro*/cell-based research, reviews, meta-analyses, duplicates, case reports, or letters. For studies using overlapping datasets, only the most recent publication was included.

### 2.3 Data extraction

Two researchers independently extracted data from full-text articles, including the following variables: first author, publication year, NCT number, median age, study design, sample size, intervention regimen, pCR, R0 resection rate, MPR, DCR, lymph node downstaging to ypN0 rate, incidence of grade ≥3 adverse events, postoperative complications, and overall TNM downstaging rate. Data not reported in the original studies were marked as “NR.” Discrepancies in data interpretation were resolved through discussion.

### 2.4 Statistical analysis

This meta-analysis was performed using R software (version 4.4.2) with the “meta” package. Effect sizes were pooled via the inverse-variance weighting method. Proportions near 0 or 1 were adjusted using the Freeman-Tukey double arcsine transformation. Heterogeneity was assessed using *χ*^2^ and *Q* tests (*I*^2^ statistic), with statistical significance set at *p* < 0.05. Given the predominance of single-arm trials and anticipated heterogeneity in participant characteristics across studies, a random-effects model was selected over a fixed-effects model. Heterogeneity thresholds followed Cochrane Handbook (11) guidelines: *I*^2^ = 0–40% (negligible), 30–60% (moderate), 50–90% (substantial), and 75–100% (considerable). Subgroup analyses were conducted to investigate sources of considerable heterogeneity identified in pooled results. Publication bias was evaluated using Egger’s test, Begg’s test, and funnel plots. Sensitivity analysis was not performed due to the predominance of single-arm studies in the included literature.

### 2.5 Assessment of study quality and publication Bias

As most included studies were single-arm clinical trials, methodological quality was assessed using the Methodological Index for Non-Randomized Studies (MINORS) scale (11). Studies scoring ≥13 points were classified as high quality, 10–12 points as moderate quality, and ≤9 points as low quality. Two researchers independently evaluated study quality, and discrepancies were resolved through discussion with a third reviewer.

## 3 Literature search results

The initial search identified 1,773 studies. After removing 473 duplicates, 1,300 studies underwent title and abstract screening, excluding 1,249 articles. Full-text review of the remaining 63 studies led to the inclusion of 12 articles that met the eligibility criteria for this meta-analysis.

**Figure 1:**
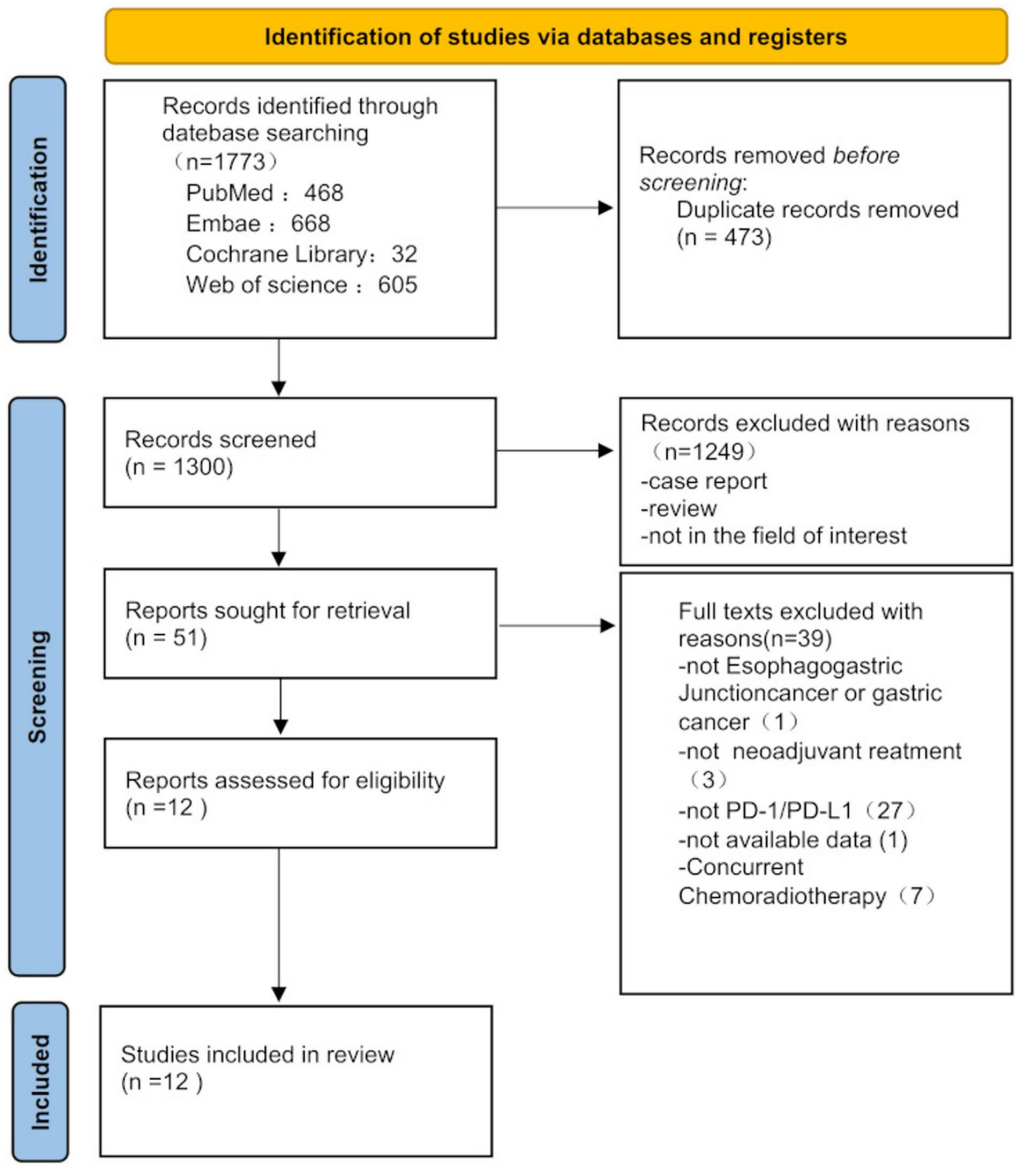
Explicitly described in the Literature Search Results section as the “search flowchart” detailing study inclusion/exclusion (1,773 records screened → 12 included studies).

Among the 12 studies, 9 were single-arm trials and 3 were retrospective studies. The primary PD-1/PD-L1 inhibitors evaluated included tislelizumab, camrelizumab, durvalumab, atezolizumab, sintilimab, and pembrolizumab. Detailed study characteristics are summarized in Table 1, and the search flowchart is shown in Figure 1. The pooled cohort comprised 428 patients, with 406 undergoing surgical intervention. Of these, 378 patients (93.1%) achieved R0 resection.

**Table 1:** Main characteristics of included studies.

Author year	Region	NCT number	Median age	Intervention model	No. of patient	Tumor type	pCR
Bao ZH 2024	China	NR	NR	NR	28	GC	4/28
Gulam A. M, 2023	USA	NCT02918162	65.5 years	pembrolizumab	36	G/GEJ	7/34
Honghai Guo2022	China	ChiCTR2000030414	62 years	Sintilimab	30	GC	10/30
H.-D. Kim, 2023	South Korea	04221555	NR	Durvalumab	40	G/GEJ	9/31
Jiang H, et al. 2022	China	NCT04065282	65.5 years	Sintilimab	36	G/GEJ	7/36
Jiang Q, 2023	China	NCT04890392	NR	tislelizumab	50	GC	13/50
Li, N 2024	China	NCT04341857	58 years	sintilimab	32	G/GEJ	5/29
Verschoor, Y L 2024	NR	NCT03448835	62 years	atezolizumab	21	G/GEJ	9/20
Xuchen Zhang 2023	China	NR	60.1 years	NR	34	GC	8/34
Yin Y, 2022	China	NCT04890392	60.5 years	tislelizumab	32	G/GEJ	8/30
Zhao, Yuzhou2024	China	NCT03939962	58 years	camrelizumab	60	GC	5/55
Zhong, WJ, 2024	China	NCT05602935	62 years	camrelizumab	29	G/GEJ	3/29

Author year	R0	MPR	DCR	ypN0 rate	trAEs	irAEs	Postoperative complications	Tumor downstaging
Bao ZH 2024	23/28	NR	NR	11/28	NR	NR	NR	NR
Gulam A. M, 2023	27/28	NR	NR	22/28	20/34	12/34	NR	NR
Honghai Guo2022	30/30	19/30	30/30	19/30	27/30	NR	NR	NR
H.-D. Kim, 2023	NR	NR	NR	NR	NR	NR	NR	NR
Jiang H, et al. 2022	35/36	17/36	36/36	21/36	10/36	3/36	7/36	27/36
Jiang Q, 2023	50/50	NR	NR	37/50	NR	NR	13/50	NR
Li, N 2024	27/29	16/29	31/32	16/29	19/32	6/32	15/29	19/29
Verschoor, Y L 2024	19/29	14/20	NR	NR	4/20	2/20	11/20	13/20
Xuchen Zhang 2023	33/34	13/34	NR	NR	10/34	NR	NR	22/34
Yin Y, 2022	30/30	17/30	29/30	4/32	NR	NR	NR	21/32
Zhao, Yuzhou2024	51/55	NR	NR	30/55	NR	NR	NR	37/55
Zhong, WJ, 2024	28/29	20/29	29/29	20/29	NR	NR	3/29	25/29

pCR, Pathological Complete Response; R0, Resection with Negative Margins; MPR, Major Pathological Response; DCR, Disease Control Rate; trAEs, Treatment-Related Adverse Events; irAEs, Immune-Related Adverse Events; ypN0 Rate, Rate of Downstaging to ypN0; Postoperative Complications, the incidence of postoperative complications.

## 4 Results

### 4.1 Primary outcomes

#### 4.1.1 Pathological complete response rate (pCR)

**Figure 2:**
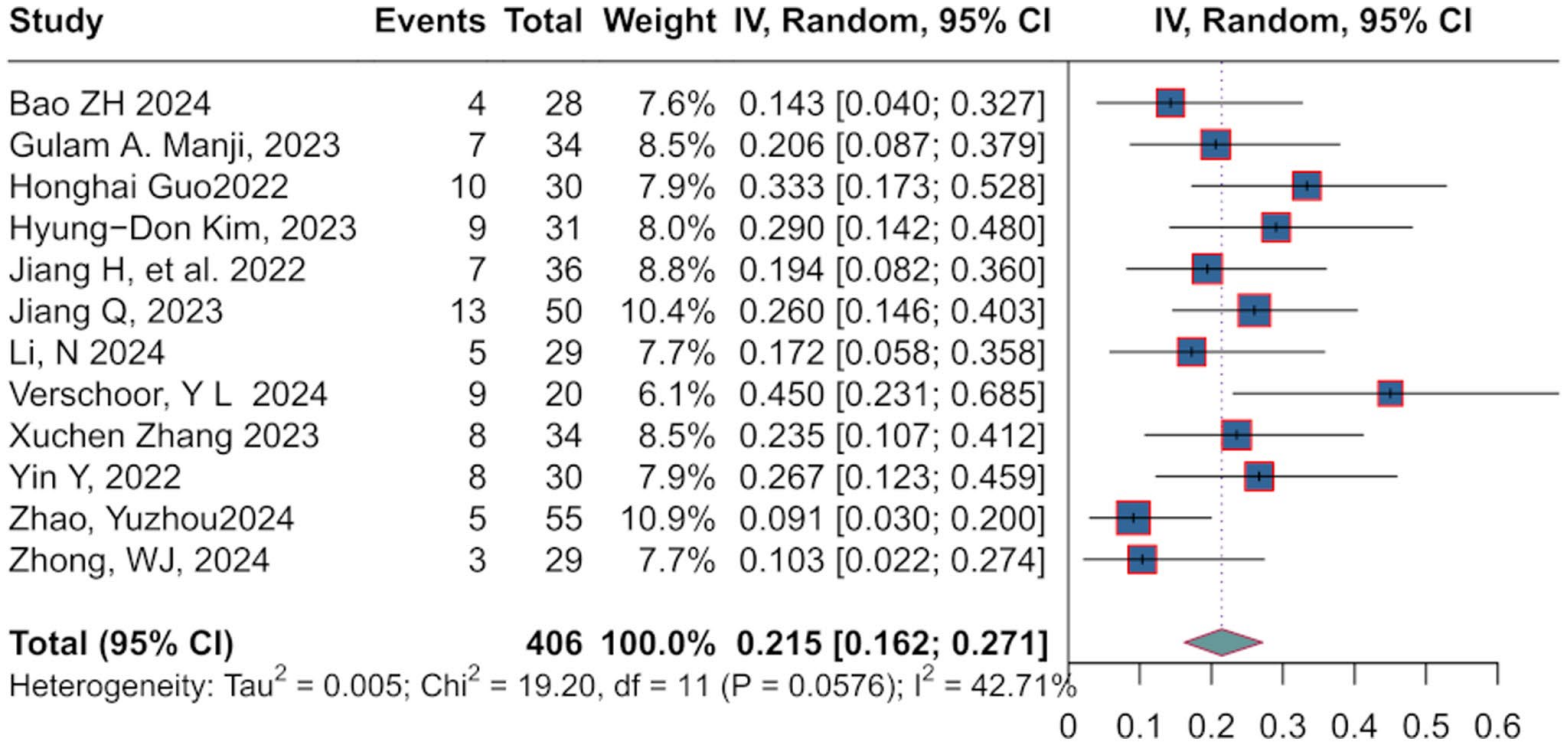
Directly referenced in the Pathological Complete Response Rate results (pCR analysis with I² = 42.71%).

Pathological complete response (pCR) is defined as the absence of residual cancer cells in both the primary tumor and lymph nodes after treatment, confirmed by postoperative pathological examination. Patients achieving pCR demonstrate prolonged overall survival and disease-free survival (12), with 5-year survival rates for certain cancers improving to 60–70% (13). Additionally, pCR serves as a surrogate endpoint in clinical trials to guide subsequent therapies and expedite drug approvals. The pooled pCR rate was 21.5% [95%:0.162; 0.271], with moderate heterogeneity (*I*^2^ = 42.71%, *p* = 0.0576) (Figure 2). A random-effects model was selected based on the observed heterogeneity levels.

#### 4.1.2 Major pathological response (MPR)

**Figure 3:**
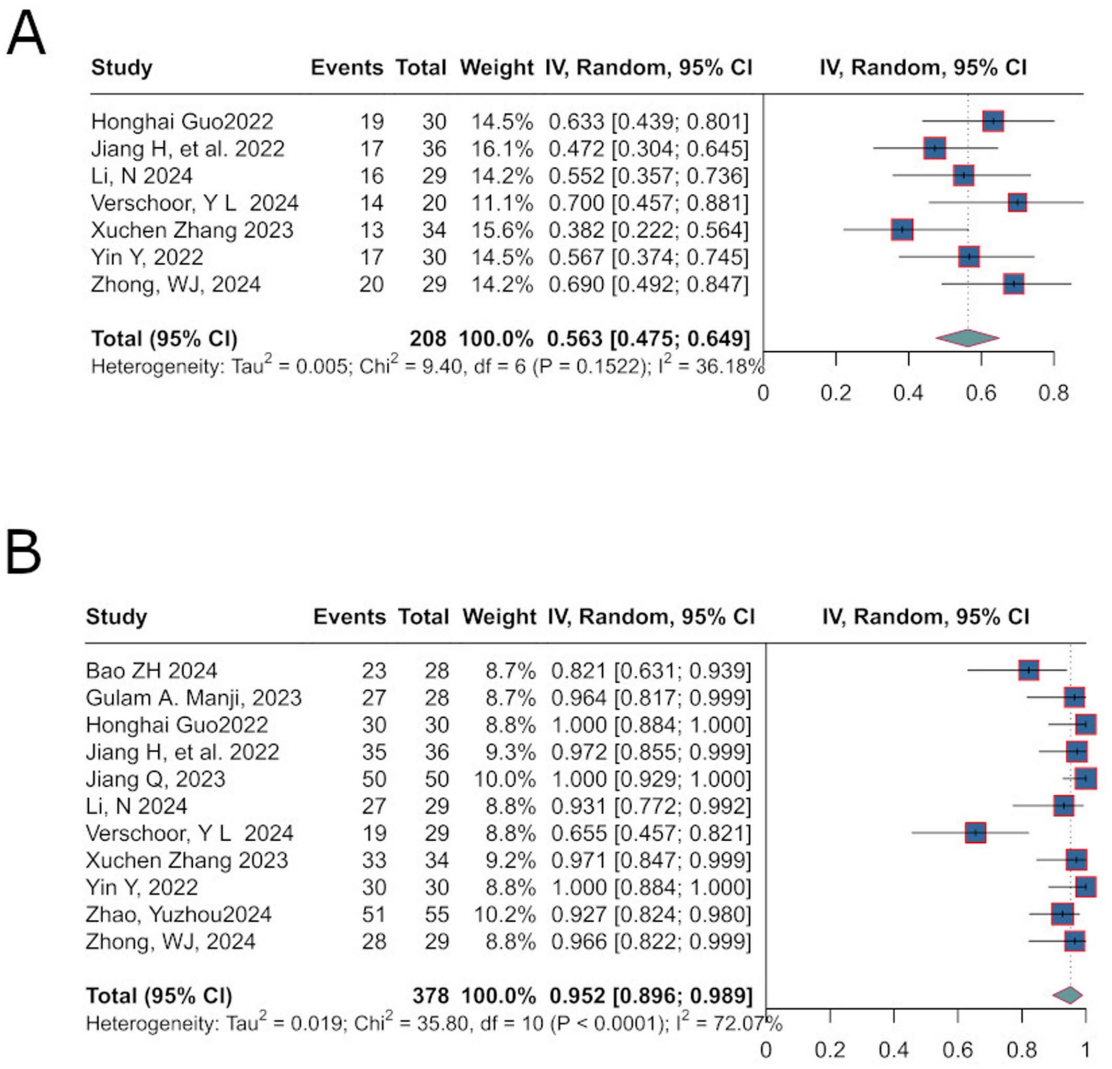
Matches subplots described in Major Pathological Response **(A)** and R0 Resection Rate **(B)** results.

Major Pathological Response (MPR) is defined as a significant reduction in the proportion of residual viable tumor cells following neoadjuvant therapy (14). The analysis revealed an effect size (ES) of 56.3% [95%CI: 0.475; 0.649] (Figure 3A), with heterogeneity results of (*p* = 0.1522; *I*^2^ = 36.18%).

#### 4.1.3 R0 resection rate

R0 resection rate refers to the complete removal of a malignant tumor with microscopically negative margins, indicating no residual cancer cells at the surgical site (15). The pooled effect size for R0 resection was 95.2% [95% CI: 0.896; 0.989] (Figure 3B). Significant heterogeneity was observed (*p* < 0.0001; *I*^2^ = 72.07%), prompting the use of a random-effects model.

### 4.2 Secondary outcomes

#### 4.2.1 Disease control rate (DCR)

**Figure 4:**
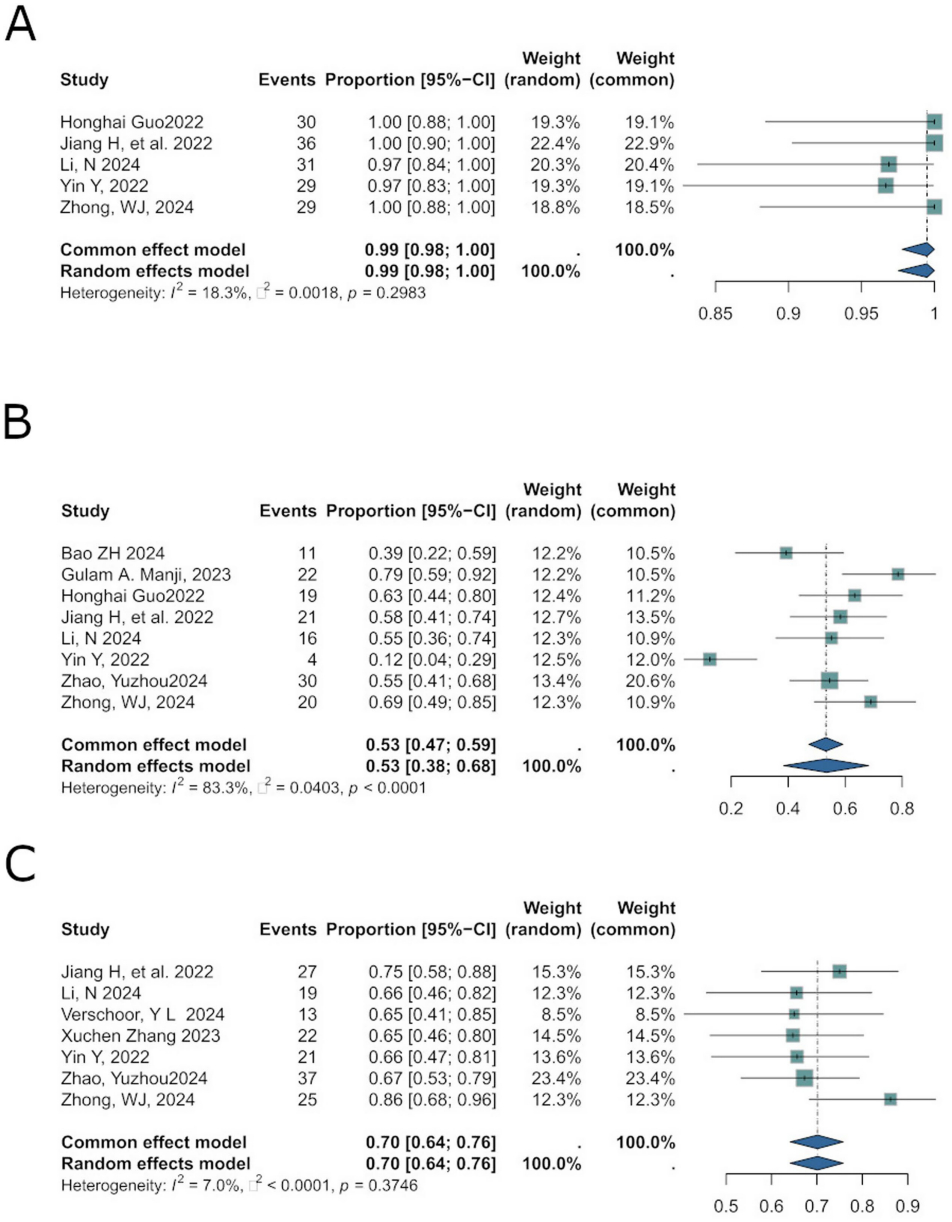
Aligns with secondary outcomes in results: DCR **(A)**, lymph node downstaging **(B)**, and overall tumor downstaging **(C)**.

Disease Control Rate (DCR) evaluates the short-term efficacy of antitumor therapies in halting disease progression. The pooled DCR effect size was 0.99 [0.98; 1.00] (Figure 4A), with low heterogeneity (*I*^2^ = 18.3%, *p* = 0.2983). A random-effects model was applied.

#### 4.2.2 Lymph node downstaging to ypN0

Lymph node downstaging to ypN0 indicates the disappearance of metastatic lymph nodes post-treatment, assessed pathologically or radiologically, which correlates with improved prognosis (16). Among 7 studies reporting this outcome, the pooled effect size was 0.53 [95%CI: 0.38; 0.68] (Figure 4B), with substantial heterogeneity (*I*^2^ = 83.3%, *p* < 0.0001). A random-effects model was used.

#### 4.2.3 Overall tumor downstaging rate

Overall tumor downstaging reflects the reduction in clinical tumor stage following neoadjuvant therapies (chemotherapy, radiotherapy, targeted therapy, or immunotherapy), facilitating surgical resection and improving prognosis. The pooled effect size was 0.70 [95%CI: 0.64; 0.76] (Figure 4C), with negligible heterogeneity (*I*^2^ = 7.0%, *p* = 0.3746).

#### 4.2.4 Treatment-related adverse events (trAEs)

**Figure 5:**
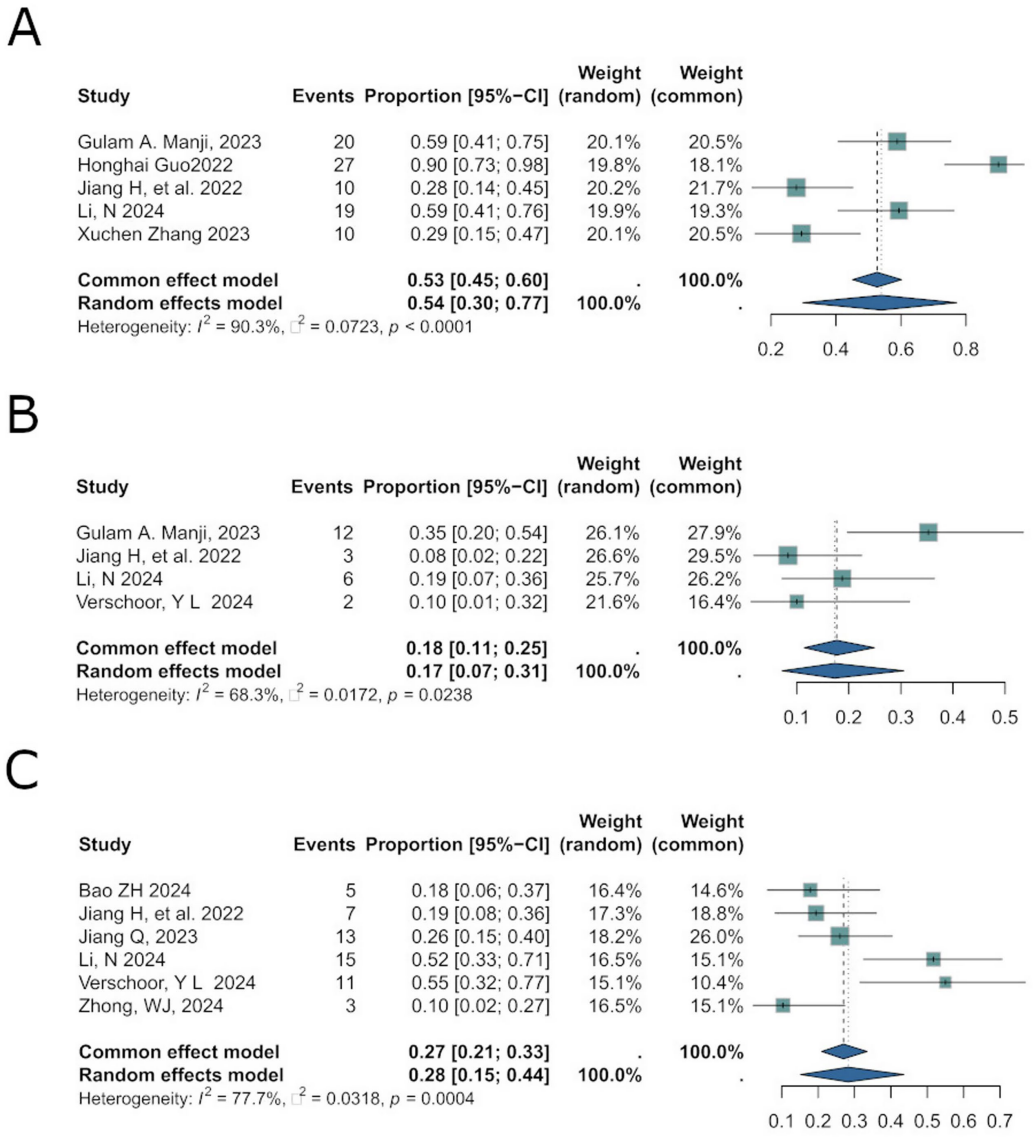
Corresponds to safety analyses for trAEs **(A)**, irAEs **(B)**, and postoperative complications **(C)** in results.

Grade ≥3 treatment-related adverse events (trAEs) are severe side effects directly attributable to therapeutic interventions, critical for evaluating treatment safet (17). The pooled incidence rate was 0.54 [95%CI: 0.30; 0.77] (Figure 5A), with significant heterogeneity (*I*^2^ = 90.3%, *p* < 0.0001).

#### 4.2.5 Immune-related adverse events (irAEs)

Immune-related adverse events (irAEs) are critical markers of toxicity risk associated with immune checkpoint inhibitors (18). Across 6 studies (total *N* = 186), the pooled incidence of irAEs was 0.17 [95% CI: 0.07; 0.31] (Figure 5B), with moderate heterogeneity (*I*^2^ = 68.3%, *p* = 0.0238). A random-effects model was employed.

#### 4.2.6 Postoperative complications

Postoperative complications, including leukopenia, neutropenia, and hemoglobin reduction, are adverse events occurring during or after surgery that may prolong recovery or threaten survival. The pooled odds ratio (OR) for complications was 0.28 [95%CI: 0.15; 0.44] (Figure 5C), with substantial heterogeneity (*I*^2^ = 77.7%, *p* = 0.0004). A random-effects model was selected.

### 4.3 Sensitivity analysis

Sensitivity analysis was performed by sequentially removing each included study to ensure the final pooled results were not significantly influenced by any single trial. During the pCR analysis, Verschoor, Y L 2024 was identified as the primary contributor to heterogeneity. Removing this study reduced heterogeneity (*I*^2^ = 31.3%). However, the remaining 11 studies still demonstrated a significant treatment effect (0.20 [95% CI: 0.15; 0.25]). In the DCR sensitivity analysis, removing Yin Y, 2022 (*I*^2^ = 0.8%) and Li, N 2024 (*I*^2^ = 2.4%) significantly reduced heterogeneity, while the overall effect estimate remained largely unchanged. The heterogeneity observed for MPR was primarily attributed to Xuchen Zhang 2023; removing this study resulted in a significant reduction in heterogeneity (*I*^2^ = 0%). For Lymph Node Downstaging to ypN0, heterogeneity was primarily associated with Yin Y, 2022; its removal led to a significant reduction in heterogeneity (*I*^2^ = 48.4%), and the effect size showed no substantial change. Conclusion: The sensitivity analysis results continue to support the efficacy and safety of combining neoadjuvant PD-1/PD-L1 inhibitors with chemotherapy.

### 4.4 Exploratory subgroup analysis

#### 4.4.1 Subgroup analysis by PD-1 inhibitor type

Among the 10 studies included, 3 used sintilimab, 2 tislelizumab, 2 camrelizumab, 1 atezolizumab, 1 durvalumab, and 1 pembrolizumab. Subgroup analyses were performed to evaluate potential differences in efficacy and safety across PD-1 inhibitor types, focusing on key outcomes such as pCR and R0 resection rates. Significant differences in pCR rates were observed among PD-1 inhibitor subtypes (*p* = 0.0051). No significant variations were detected in MPR, DCR, or lymph node downstaging to ypN0 rates.

#### 4.4.2 Gastric cancer (GC) group vs. mixed gastric/esophagogastric junction cancer (GC/EGJC) group

To investigate potential heterogeneity based on anatomical tumor location, we stratified the included studies into two subgroups: a Gastric Cancer (GC) group and a Mixed Gastric/Esophagogastric Junction Cancer (GC/EGJC) group. Subgroup analysis revealed no statistically significant differences in rates of pathological complete response (pCR) (*p* = 0.6715) or R0 resection (*p* = 0.5829) between these groups.

#### 4.4.3 PD-1 inhibitor vs. PD-L1 inhibitor subgroups

Subgroup analyses compared PD-1 inhibitors (*n* = 8 studies) and PD-L1 inhibitors (Verschoor YL 2024, Kim HD 2023). The PD-L1 subgroup showed a higher pooled pCR rate (0.21 [95% CI: 0.16; 0.27]) than the PD-1 subgroup (0.19 [95% CI: 0.15; 0.25]), with significant inter-subgroup heterogeneity (*χ*^2^ = 4.01, df = 1; *p* = 0.0453). Postoperative complication rates were also higher in the PD-L1 subgroup (0.31 [95% CI: 0.15; 0.49]) compared to the PD-1 subgroup (0.26 [95% CI:0.11; 0.43]; *χ*^2^ = 4.27, df = 1; *p* = 0.0389). No significant differences were observed in grade ≥3 adverse events, irAEs, MPR, or tumor downstaging rates. These findings suggest that therapeutic efficacy may vary by target (PD-1 vs. PD-L1), though overall safety profiles remain comparable.

## 5 Discussion

Over the past decades, therapeutic advancements in gastric cancer (GC) have expanded treatment options, including perioperative chemotherapy and adjuvant chemoradiotherapy, which reduce recurrence risks and improve survival. Immune checkpoint inhibitors (ICIs) and targeted therapies are now incorporated into clinical guidelines globally. For instance, HER2-targeted therapies have improved objective response rates (ORR: 47.3% vs. 34.5%) and progression-free survival (PFS: 6.7 vs. 5.5 months; HR: 0.71, 95% CI: 0.59–0.85; *p* < 0.0002) (19). ICIs, particularly PD-1/PD-L1 inhibitors, have shown promise in untreated advanced or metastatic GC patients with high microsatellite instability (MSI-H) or mismatch repair deficiency (dMMR) (20), offering new hope for unresectable cases. Unlike conventional therapies, immunotherapy exerts antitumor effects by activating the host immune system and reversing immune suppression. Emerging evidence suggests that immunotherapy can potentiate T-cell responses against tumor antigens while enhancing the detection and elimination of micrometastatic deposits beyond the resected tumor margins. When combined with chemotherapeutic agents (e.g., paclitaxel, platinum compounds), it may induce immunogenic cell death (ICD), triggering the release of damage-associated molecular patterns (DAMPs) that enhance antigen presentation by APCs. This mechanism synergizes with PD-1/PD-L1 inhibitors to achieve superior therapeutic efficacy. Notably, PD-1 inhibitors are now approved for unresectable/metastatic GC in several countries, despite incomplete mechanistic understanding (21).

**Treatment efficacy**: The pooled pCR rate of 21.5% [0.162; 0.271] observed in this meta-analysis exceeds the 14.3% pCR reported in the POET trial for esophagogastric adenocarcinoma and approaches the 23% rate in the CROSS study (22). Notably, Verschoor YL 2024 achieved a pCR of 45%. The mean MPR rate was 56.3% [0.475; 0.649], with 3 studies reporting MPR > 60%, including a remarkable 70% in Verschoor YL 2024.

**Safety profile**: The pooled R0 resection rate of 95.2% [95% CI: 0.896; 0.989] surpasses rates from RESONANCE (73.1%) (19, 20), CROSS (82–85%) (22), and FLOT4-AIO (84%) (23), suggesting enhanced tolerability of PD-1/PD-L1 inhibitor-based neoadjuvant regimens.

**Limitations**: Furthermore, this study has several limitations. First, the analyzed endpoints were short-term. Moreover, due to a lack of reported data in the included studies, critical long-term outcomes such as overall survival (OS), disease-free survival (DFS), and the incidence of adverse immune reactions were not assessable in this analysis. This omission may introduce bias regarding the long-term survival impact of this treatment regimen. Second, the overall sample size was limited, comprising only 428 patients (406 underwent surgical intervention), with merely 88 patients achieving a pathological complete response (pCR). This small sample size, particularly the low number of pCR events, results in limited statistical power. It is important to note that treatment efficacy varies significantly across different tumor types. Unfortunately, the studies included in our analysis lacked sufficient data to perform further subgroup analyses based on tumor type. Additionally, since most of the included studies were single-arm trials without control groups, residual confounding factors may have led to an overestimation of the treatment effect. Furthermore, the risk of funnel plot asymmetry is heightened due to potential publication bias, as single-arm studies with positive results are more likely to be published, while long-term negative outcomes may remain unreported.

**Future directions**: Additionally, while the study results demonstrated encouraging outcomes for R0 resection rates with neoadjuvant PD-1/PD-L1 inhibitor therapy, the analysis was limited by the absence of high-quality randomized controlled trials (RCTs). Among the 12 included studies, 9 were single-arm trials and 3 were retrospective analyses. Single-arm trials cannot exclude time effects or selection bias, making it difficult to establish causal relationships. Furthermore, substantial heterogeneity existed in treatment protocols: the specific PD-1/PD-L1 inhibitors used, chemotherapy regimens, and treatment cycles were not standardized. The included patients also encompassed varying disease stages. This variability complicates the interpretation of whether these factors influenced treatment efficacy or safety. Concurrently, the lack of standardized biomarker testing and stratified study designs within the included literature represents a missed opportunity for identifying predictive biomarkers of response. Therefore, future research should focus on determining optimal neoadjuvant PD-1/PD-L1 inhibitor-based regimens to maximize rates of pathological complete response (pCR) and overall survival (OS). Key priorities include defining the optimal treatment duration and timing of surgery, and incorporating biomarker stratification (e.g., PD-L1 Combined Positive Score (CPS), Microsatellite Instability (MSI) status). The integration of biomarker-driven patient stratification will be pivotal in future clinical practice, offering significant improvements in objective response rates (ORR) and survival outcomes while minimizing ineffective treatments and associated toxicities. For example, PD-L1-negative patients, who typically exhibit response rates below 10%, could avoid unnecessary immunotherapy, thereby reducing financial burdens and the risk of immune-related adverse events (irAEs). Conversely, MSI-H patients may derive greater benefit from immunotherapy alone or de-escalated regimens, circumventing the overt use of chemotherapy (24, 25). Prospective trials should incorporate predictive biomarker analyses to validate potential signatures.

## Data Availability

The original contributions presented in the study are included in the article/supplementary material, further inquiries can be directed to the corresponding author.
