# Supplementary material for: Neoadjuvant PD-1/PD-L1 inhibitors combined with chemotherapy in gastric cancer and gastroesophageal junction adenocarcinoma: a systematic review and meta-analysis of single-arm studies
Source: Front Med (Lausanne). 2025 Aug 5;12:1625259. doi: 10.3389/fmed.2025.1625259 (PMC12361224; doi:10.3389/fmed.2025.1625259)
Supplement: Supplementary file 1 [file Table_1.docx]

Supplementary Material

1. **Search strategies**
   1. Search strategy for MEDLINE via PubMed.

("Stomach Neoplasms"[MeSH Terms] OR ("neoplasm stomach"[Title/Abstract] OR "stomach neoplasm"[Title/Abstract] OR "neoplasms stomach"[Title/Abstract] OR "gastric neoplasms"[Title/Abstract] OR "gastric neoplasm"[Title/Abstract] OR "neoplasm gastric"[Title/Abstract] OR "neoplasms gastric"[Title/Abstract] OR "cancer of stomach"[Title/Abstract] OR "stomach cancers"[Title/Abstract] OR "gastric cancer"[Title/Abstract] OR "cancer gastric"[Title/Abstract] OR "cancers gastric"[Title/Abstract] OR "gastric cancers"[Title/Abstract] OR "stomach cancer"[Title/Abstract] OR "cancer stomach"[Title/Abstract] OR "cancers stomach"[Title/Abstract] OR "cancer of the stomach"[Title/Abstract] OR (("Stomach Neoplasms"[MeSH Terms] OR ("Stomach"[All Fields] AND "Neoplasms"[All Fields]) OR "Stomach Neoplasms"[All Fields] OR ("Gastric"[All Fields] AND "Cancer"[All Fields]) OR "gastric cancer"[All Fields]) AND "familial diffuse"[Title/Abstract]))) AND ("Neoadjuvant Therapy"[MeSH Terms] OR ("neoadjuvant therapies"[Title/Abstract] OR "undefined"[All Fields] OR "therapy neoadjuvant"[Title/Abstract] OR "neoadjuvant treatments"[Title/Abstract] OR "neoadjuvant treatment"[Title/Abstract] OR "treatment neoadjuvant"[Title/Abstract] OR "neoadjuvant chemoradiotherapy"[Title/Abstract] OR "chemoradiotherapy neoadjuvant"[Title/Abstract] OR "neoadjuvant chemoradiotherapies"[Title/Abstract] OR "neoadjuvant chemoradiation therapy"[Title/Abstract] OR "chemoradiation therapy neoadjuvant"[Title/Abstract] OR (("neoadjuvancy"[All Fields] OR "Neoadjuvant Therapy"[MeSH Terms] OR ("Neoadjuvant"[All Fields] AND "Therapy"[All Fields]) OR "Neoadjuvant Therapy"[All Fields] OR "Neoadjuvant"[All Fields] OR "neoadjuvants"[All Fields] OR "neoadjuvent"[All Fields]) AND "chemoradiation therapies"[Title/Abstract]) OR (("therapeutics"[MeSH Terms] OR "therapeutics"[All Fields] OR "Therapies"[All Fields] OR "Therapy"[MeSH Subheading] OR "Therapy"[All Fields] OR "therapy s"[All Fields] OR "therapys"[All Fields]) AND "neoadjuvant chemoradiation"[Title/Abstract]) OR "neoadjuvant chemoradiation treatment"[Title/Abstract] OR (("Chemoradiotherapy"[MeSH Terms] OR "Chemoradiotherapy"[All Fields] OR "Chemoradiation"[All Fields]) AND "treatment neoadjuvant"[Title/Abstract]) OR (("neoadjuvancy"[All Fields] OR "Neoadjuvant Therapy"[MeSH Terms] OR ("Neoadjuvant"[All Fields] AND "Therapy"[All Fields]) OR "Neoadjuvant Therapy"[All Fields] OR "Neoadjuvant"[All Fields] OR "neoadjuvants"[All Fields] OR "neoadjuvent"[All Fields]) AND "chemoradiation treatments"[Title/Abstract]) OR "treatment neoadjuvant chemoradiation"[Title/Abstract] OR "neoadjuvant chemoradiation"[Title/Abstract] OR "chemoradiation neoadjuvant"[Title/Abstract] OR "neoadjuvant radiotherapy"[Title/Abstract] OR (("neoadjuvancy"[All Fields] OR "Neoadjuvant Therapy"[MeSH Terms] OR ("Neoadjuvant"[All Fields] AND "Therapy"[All Fields]) OR "Neoadjuvant Therapy"[All Fields] OR "Neoadjuvant"[All Fields] OR "neoadjuvants"[All Fields] OR "neoadjuvent"[All Fields]) AND "Radiotherapies"[Title/Abstract]) OR "radiotherapy neoadjuvant"[Title/Abstract] OR "neoadjuvant radiation treatment"[Title/Abstract] OR (("neoadjuvancy"[All Fields] OR "Neoadjuvant Therapy"[MeSH Terms] OR ("Neoadjuvant"[All Fields] AND "Therapy"[All Fields]) OR "Neoadjuvant Therapy"[All Fields] OR "Neoadjuvant"[All Fields] OR "neoadjuvants"[All Fields] OR "neoadjuvent"[All Fields]) AND "radiation treatments"[Title/Abstract]) OR (("radiate"[All Fields] OR "radiated"[All Fields] OR "radiates"[All Fields] OR "radiating"[All Fields] OR "Radiation"[MeSH Terms] OR "Radiation"[All Fields] OR "electromagnetic radiation"[MeSH Terms] OR ("electromagnetic"[All Fields] AND "Radiation"[All Fields]) OR "electromagnetic radiation"[All Fields] OR "Radiations"[All Fields] OR "radiation s"[All Fields] OR "radiator"[All Fields] OR "radiators"[All Fields]) AND "treatment neoadjuvant"[Title/Abstract]) OR (("therapeutics"[MeSH Terms] OR "therapeutics"[All Fields] OR "Treatments"[All Fields] OR "Therapy"[MeSH Subheading] OR "Therapy"[All Fields] OR "Treatment"[All Fields] OR "treatment s"[All Fields]) AND "neoadjuvant radiation"[Title/Abstract]) OR "neoadjuvant radiation therapy"[Title/Abstract] OR (("neoadjuvancy"[All Fields] OR "Neoadjuvant Therapy"[MeSH Terms] OR ("Neoadjuvant"[All Fields] AND "Therapy"[All Fields]) OR "Neoadjuvant Therapy"[All Fields] OR "Neoadjuvant"[All Fields] OR "neoadjuvants"[All Fields] OR "neoadjuvent"[All Fields]) AND "radiation therapies"[Title/Abstract]) OR "radiation therapy neoadjuvant"[Title/Abstract] OR (("therapeutics"[MeSH Terms] OR "therapeutics"[All Fields] OR "Therapies"[All Fields] OR "Therapy"[MeSH Subheading] OR "Therapy"[All Fields] OR "therapy s"[All Fields] OR "therapys"[All Fields]) AND "neoadjuvant radiation"[Title/Abstract]) OR "neoadjuvant radiation"[Title/Abstract] OR (("neoadjuvancy"[All Fields] OR "Neoadjuvant Therapy"[MeSH Terms] OR ("Neoadjuvant"[All Fields] AND "Therapy"[All Fields]) OR "Neoadjuvant Therapy"[All Fields] OR "Neoadjuvant"[All Fields] OR "neoadjuvants"[All Fields] OR "neoadjuvent"[All Fields]) AND "Radiations"[Title/Abstract]) OR "radiation neoadjuvant"[Title/Abstract] OR "neoadjuvant chemotherapy"[Title/Abstract] OR "chemotherapy neoadjuvant"[Title/Abstract] OR "neoadjuvant chemotherapies"[Title/Abstract] OR "neoadjuvant chemotherapy treatment"[Title/Abstract] OR (("chemotherapy s"[All Fields] OR "drug therapy"[MeSH Terms] OR ("drug"[All Fields] AND "Therapy"[All Fields]) OR "drug therapy"[All Fields] OR "Chemotherapies"[All Fields] OR "drug therapy"[MeSH Subheading] OR "Chemotherapy"[All Fields]) AND "treatment neoadjuvant"[Title/Abstract]) OR "neoadjuvant chemotherapy treatments"[Title/Abstract] OR "treatment neoadjuvant chemotherapy"[Title/Abstract] OR "neoadjuvant systemic therapy"[Title/Abstract] OR "neoadjuvant systemic therapies"[Title/Abstract] OR "systemic therapy neoadjuvant"[Title/Abstract] OR (("therapeutics"[MeSH Terms] OR "therapeutics"[All Fields] OR "Therapies"[All Fields] OR "Therapy"[MeSH Subheading] OR "Therapy"[All Fields] OR "therapy s"[All Fields] OR "therapys"[All Fields]) AND "neoadjuvant systemic"[Title/Abstract]) OR "neoadjuvant systemic treatment"[Title/Abstract] OR "neoadjuvant systemic treatments"[Title/Abstract] OR "systemic treatment neoadjuvant"[Title/Abstract] OR (("therapeutics"[MeSH Terms] OR "therapeutics"[All Fields] OR "Treatments"[All Fields] OR "Therapy"[MeSH Subheading] OR "Therapy"[All Fields] OR "Treatment"[All Fields] OR "treatment s"[All Fields]) AND "neoadjuvant systemic"[Title/Abstract]))) AND ("Esophagogastric Junction"[MeSH Terms] OR (("Junction"[All Fields] OR "junction s"[All Fields] OR "junctional"[All Fields] OR "junctionally"[All Fields] OR "Junctions"[All Fields]) AND "Esophagogastric"[Title/Abstract]) OR "gastroesophageal junction"[Title/Abstract] OR "gastroesophageal junctions"[Title/Abstract] OR (("Junction"[All Fields] OR "junction s"[All Fields] OR "junctional"[All Fields] OR "junctionally"[All Fields] OR "Junctions"[All Fields]) AND "Gastroesophageal"[Title/Abstract]) OR (("Junction"[All Fields] OR "junction s"[All Fields] OR "junctional"[All Fields] OR "junctionally"[All Fields] OR "Junctions"[All Fields]) AND "Gastroesophageal"[Title/Abstract]))

- 1. Search strategy for MEDLINE via Cochrane.

MeSH descriptor: [Stomach Neoplasms] explode all trees

(Neoplasm, Stomach):ti,ab,kw OR (Stomach Neoplasm):ti,ab,kw OR (Neoplasms, Stomach):ti,ab,kw OR (Gastric Neoplasms):ti,ab,kw OR (Gastric Neoplasm):ti,ab,kw OR (Neoplasm, Gastric):ti,ab,kw OR (Neoplasms, Gastric):ti,ab,kw OR (Cancer of Stomach):ti,ab,kw OR (Stomach Cancers):ti,ab,kw OR (Gastric Cancer):ti,ab,kw OR (Cancer, Gastric):ti,ab,kw OR (Cancers, Gastric):ti,ab,kw OR (Gastric Cancers):ti,ab,kw OR (Stomach Cancer):ti,ab,kw OR (Cancer, Stomach):ti,ab,kw OR (Cancers, Stomach):ti,ab,kw OR (Cancer of the Stomach):ti,ab,kw OR (Gastric Cancer, Familial Diffuse)

MeSH descriptor: [Esophagogastric Junction] explode all trees

(Junction, Esophagogastric):ti,ab,kw OR (Gastroesophageal Junction):ti,ab,kw OR (Gastroesophageal Junctions):ti,ab,kw OR (Junction, Gastroesophageal):ti,ab,kw OR (Junctions, Gastroesophageal):ti,ab,kw

(neoadjuvant therapies):ti,ab,kw OR (therapy neoadjuvant):ti,ab,kw OR (Neoadjuvant Treatments):ti,ab,kw OR (Neoadjuvant Treatment):ti,ab,kw OR (Treatment, Neoadjuvant):ti,ab,kw OR (Neoadjuvant Chemoradiotherapy):ti,ab,kw OR (Chemoradiotherapy, Neoadjuvant):ti,ab,kw OR (Neoadjuvant Chemoradiotherapies):ti,ab,kw OR (Neoadjuvant Chemoradiation Therapy):ti,ab,kw OR (Chemoradiation Therapy, Neoadjuvant):ti,ab,kw OR (Neoadjuvant Chemoradiation Therapies):ti,ab,kw OR (Therapy, Neoadjuvant Chemoradiation):ti,ab,kw OR (Neoadjuvant Chemoradiation Treatment):ti,ab,kw OR (Chemoradiation Treatment, Neoadjuvant):ti,ab,kw OR (Neoadjuvant Chemoradiation Treatments):ti,ab,kw OR (Treatment, Neoadjuvant Chemoradiation):ti,ab,kw OR (Neoadjuvant Chemoradiation):ti,ab,kw OR (Chemoradiation, Neoadjuvant):ti,ab,kw OR (Neoadjuvant Chemoradiations):ti,ab,kw OR (Neoadjuvant Radiotherapy):ti,ab,kw OR (Neoadjuvant Radiotherapies):ti,ab,kw OR (Radiotherapy, Neoadjuvant):ti,ab,kw OR (Neoadjuvant Radiation Treatment):ti,ab,kw OR (Neoadjuvant Radiation Treatmaents):ti,ab,kw OR (Radiation Treatment, Neoadjuvant):ti,ab,kw OR (Treatment, Neoadjuvant Radiation):ti,ab,kw OR (Neoadjuvant Radiation Therapy):ti,ab,kw OR (Neoadjuvant Radiation Therapies):ti,ab,kw OR (Radiation Therapy, Neoadjuvant):ti,ab,kw OR (Therapy, Neoadjuvant Radiation):ti,ab,kw OR (Neoadjuvant Radiation):ti,ab,kw OR (Neoadjuvant Radiations):ti,ab,kw OR (Radiation, Neoadjuvant):ti,ab,kw OR (Neoadjuvant Chemotherapy):ti,ab,kw OR (Chemotherapy, Neoadjuvant):ti,ab,kw OR (Neoadjuvant Chemotherapies):ti,ab,kw OR (Neoadjuvant Chemotherapy Treatment):ti,ab,kw OR (Chemotherapy Treatment, Neoadjuvant):ti,ab,kw OR (Neoadjuvant Chemotherapy Treatments):ti,ab,kw OR (Treatment, Neoadjuvant Chemotherapy):ti,ab,kw OR (Neoadjuvant Systemic Therapy):ti,ab,kw OR (Neoadjuvant Systemic Therapies):ti,ab,kw OR (Systemic Therapy, Neoadjuvant):ti,ab,kw OR (Therapy, Neoadjuvant Systemic):ti,ab,kw OR (Neoadjuvant Systemic Treatment):ti,ab,kw OR (Neoadjuvant Systemic Treatments):ti,ab,kw OR (Systemic Treatment, Neoadjuvant):ti,ab,kw OR (Treatment, Neoadjuvant Systemic):ti,ab,kw

MeSH descriptor: [Neoadjuvant Therapy] explode all trees

- 1. Search strategy for MEDLINE via web of science.

1: TS=(Neoadjuvant Therapy OR neoadjuvant therapies OR therapy neoadjuvant OR Neoadjuvant Treatments OR Neoadjuvant Treatment OR Treatment, Neoadjuvant OR Neoadjuvant Chem ORadiotherapy OR Chem ORadiotherapy, Neoadjuvant OR Neoadjuvant Chem ORadiotherapies OR Neoadjuvant Chem ORadiation Therapy OR Chem ORadiation Therapy, Neoadjuvant OR Neoadjuvant Chem ORadiation Therapies OR Therapy, Neoadjuvant Chem ORadiation OR Neoadjuvant Chem ORadiation Treatment OR Chem ORadiation Treatment, Neoadjuvant OR Neoadjuvant Chem ORadiation Treatments OR Treatment, Neoadjuvant Chem ORadiation OR Neoadjuvant Chem ORadiation OR Chem ORadiation, Neoadjuvant OR Neoadjuvant Chem ORadiations OR Neoadjuvant Radiotherapy OR Neoadjuvant Radiotherapies OR Radiotherapy, Neoadjuvant OR Neoadjuvant Radiation Treatment OR Neoadjuvant Radiation Treatmaents OR Radiation Treatment, Neoadjuvant OR Treatment, Neoadjuvant Radiation OR Neoadjuvant Radiation Therapy OR Neoadjuvant Radiation Therapies OR Radiation Therapy, Neoadjuvant OR Therapy, Neoadjuvant Radiation OR Neoadjuvant Radiation OR Neoadjuvant Radiations OR Radiation, Neoadjuvant OR Neoadjuvant Chemotherapy OR Chemotherapy, Neoadjuvant OR Neoadjuvant Chemotherapies OR Neoadjuvant Chemotherapy Treatment OR Chemotherapy Treatment, Neoadjuvant OR Neoadjuvant Chemotherapy Treatments OR Treatment, Neoadjuvant Chemotherapy OR Neoadjuvant Systemic Therapy OR Neoadjuvant Systemic Therapies OR Systemic Therapy, Neoadjuvant OR Therapy, Neoadjuvant Systemic OR Neoadjuvant Systemic Treatment OR Neoadjuvant Systemic Treatments OR Systemic Treatment, Neoadjuvant OR Treatment, Neoadjuvant Systemic)

2: TS=(Neoplasm, Stomach OR Stomach Neoplasm OR Neoplasms, Stomach OR Gastric Neoplasms OR Gastric Neoplasm OR Neoplasm, Gastric OR Neoplasms, Gastric OR Cancer of Stomach OR Stomach Cancers OR Gastric Cancer OR Cancer, Gastric OR Cancers, Gastric OR Gastric Cancers OR Stomach Cancer OR Cancer, Stomach OR Cancers, Stomach OR Cancer of the Stomach OR Gastric Cancer, Familial Diffuse )

3: TS=(Esophagogastric Junction OR Junction, Esophagogastric OR Gastroesophageal Junction OR Gastroesophageal Junctions OR Junction, Gastroesophageal OR Junctions, Gastroesophageal)

- 1. Search strategy for MEDLINE via Embase.

#1：'stomach tumor'/exp

#2：'neoplasm, stomach':ab,kw,ti OR 'stomach neoplasm':ab,kw,ti OR 'neoplasms, stomach':ab,kw,ti OR 'gastric neoplasms':ab,kw,ti OR 'gastric neoplasm':ab,kw,ti OR 'neoplasm, gastric':ab,kw,ti OR 'neoplasms, gastric':ab,kw,ti OR 'cancer of stomach':ab,kw,ti OR 'stomach cancers':ab,kw,ti OR 'gastric cancer':ab,kw,ti OR 'cancer, gastric':ab,kw,ti OR 'cancers, gastric':ab,kw,ti OR 'gastric cancers':ab,kw,ti OR 'stomach cancer':ab,kw,ti OR 'cancer, stomach':ab,kw,ti OR 'cancers, stomach':ab,kw,ti OR 'cancer of the stomach':ab,kw,ti OR 'gastric cancer, familial diffuse':ab,kw,ti

#3：'gastroesophageal junction'/exp OR 'gastroesophageal junction'

#4:'junction, esophagogastric':ab,kw,ti OR 'gastroesophageal junction':ab,kw,ti OR 'gastroesophageal junctions':ab,kw,ti OR 'junction, gastroesophageal':ab,kw,ti OR 'junctions, gastroesophageal':ab,kw,ti

#5：'neoadjuvant therapy'/exp OR 'neoadjuvant therapy'

#6：'neoadjuvant therapies':ab,kw,ti OR 'therapy neoadjuvant':ab,kw,ti OR 'neoadjuvant treatments':ab,kw,ti OR 'neoadjuvant treatment':ab,kw,ti OR 'treatment, neoadjuvant':ab,kw,ti OR 'neoadjuvant chemoradiotherapy':ab,kw,ti OR 'chemoradiotherapy, neoadjuvant':ab,kw,ti OR 'neoadjuvant chemoradiotherapies':ab,kw,ti OR 'neoadjuvant chemoradiation therapy':ab,kw,ti OR 'chemoradiation therapy, neoadjuvant':ab,kw,ti OR 'neoadjuvant chemoradiation therapies':ab,kw,ti OR 'therapy, neoadjuvant chemoradiation':ab,kw,ti OR 'neoadjuvant chemoradiation treatment':ab,kw,ti OR 'chemoradiation treatment, neoadjuvant':ab,kw,ti OR 'neoadjuvant chemoradiation treatments':ab,kw,ti OR 'treatment, neoadjuvant chemoradiation':ab,kw,ti OR 'neoadjuvant chemoradiation':ab,kw,ti OR 'chemoradiation, neoadjuvant':ab,kw,ti OR 'neoadjuvant chemoradiations':ab,kw,ti OR 'neoadjuvant radiotherapy':ab,kw,ti OR 'neoadjuvant radiotherapies':ab,kw,ti OR 'radiotherapy, neoadjuvant':ab,kw,ti OR 'neoadjuvant radiation treatment':ab,kw,ti OR 'neoadjuvant radiation treatmaents':ab,kw,ti OR 'radiation treatment, neoadjuvant':ab,kw,ti OR 'treatment, neoadjuvant radiation':ab,kw,ti OR 'neoadjuvant radiation therapy':ab,kw,ti OR 'neoadjuvant radiation therapies':ab,kw,ti OR 'radiation therapy, neoadjuvant':ab,kw,ti OR 'therapy, neoadjuvant radiation':ab,kw,ti OR 'neoadjuvant radiation':ab,kw,ti OR 'neoadjuvant radiations':ab,kw,ti OR 'radiation, neoadjuvant':ab,kw,ti OR 'neoadjuvant chemotherapy':ab,kw,ti OR 'chemotherapy, neoadjuvant':ab,kw,ti OR 'neoadjuvant chemotherapies':ab,kw,ti OR 'neoadjuvant chemotherapy treatment':ab,kw,ti OR 'chemotherapy treatment, neoadjuvant':ab,kw,ti OR 'neoadjuvant chemotherapy treatments':ab,kw,ti OR 'treatment, neoadjuvant chemotherapy':ab,kw,ti OR 'neoadjuvant systemic therapy':ab,kw,ti OR 'neoadjuvant systemic therapies':ab,kw,ti OR 'systemic therapy, neoadjuvant':ab,kw,ti OR 'therapy, neoadjuvant systemic':ab,kw,ti OR 'neoadjuvant systemic treatment':ab,kw,ti OR 'neoadjuvant systemic treatments':ab,kw,ti OR 'systemic treatment, neoadjuvant':ab,kw,ti OR 'treatment, neoadjuvant systemic':ab,kw,ti

#1 AND #2 AND #3 AND #4 AND #5 AND #6

# Supplementary Figures and Tables

## Supplementary Figures


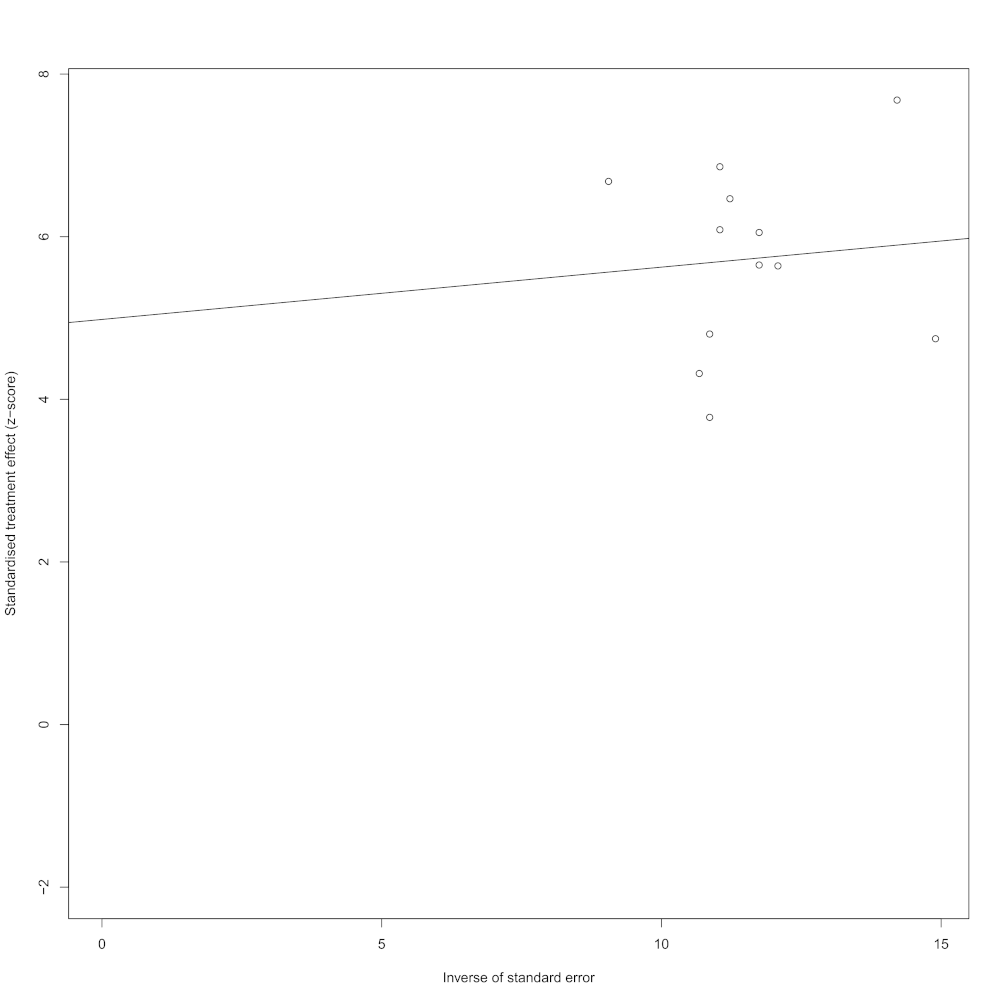


Figure 1 Egger test plot for pCR

**Supplementary Figure 1.** Egger test plot for pCR


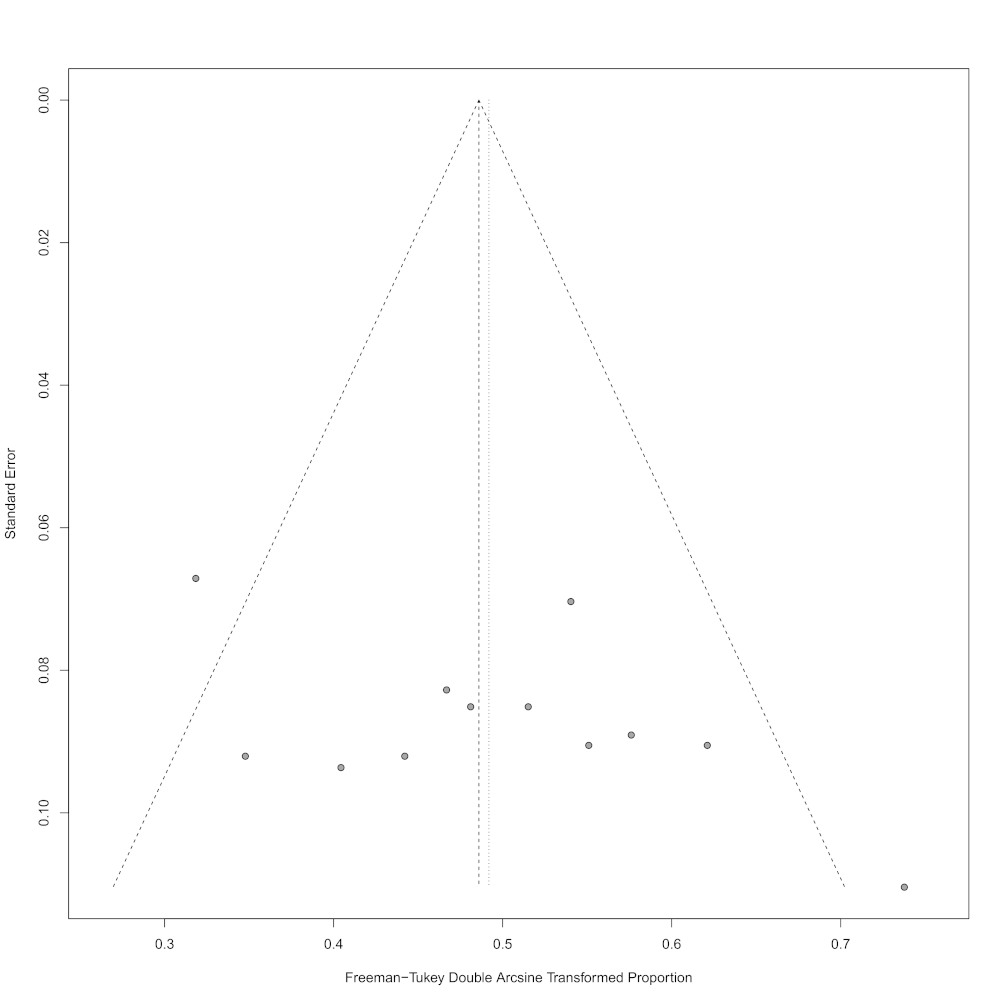


Figure 2 Funnel plot for pCR

**Supplementary Figure 2.** Funnel plot for pCR


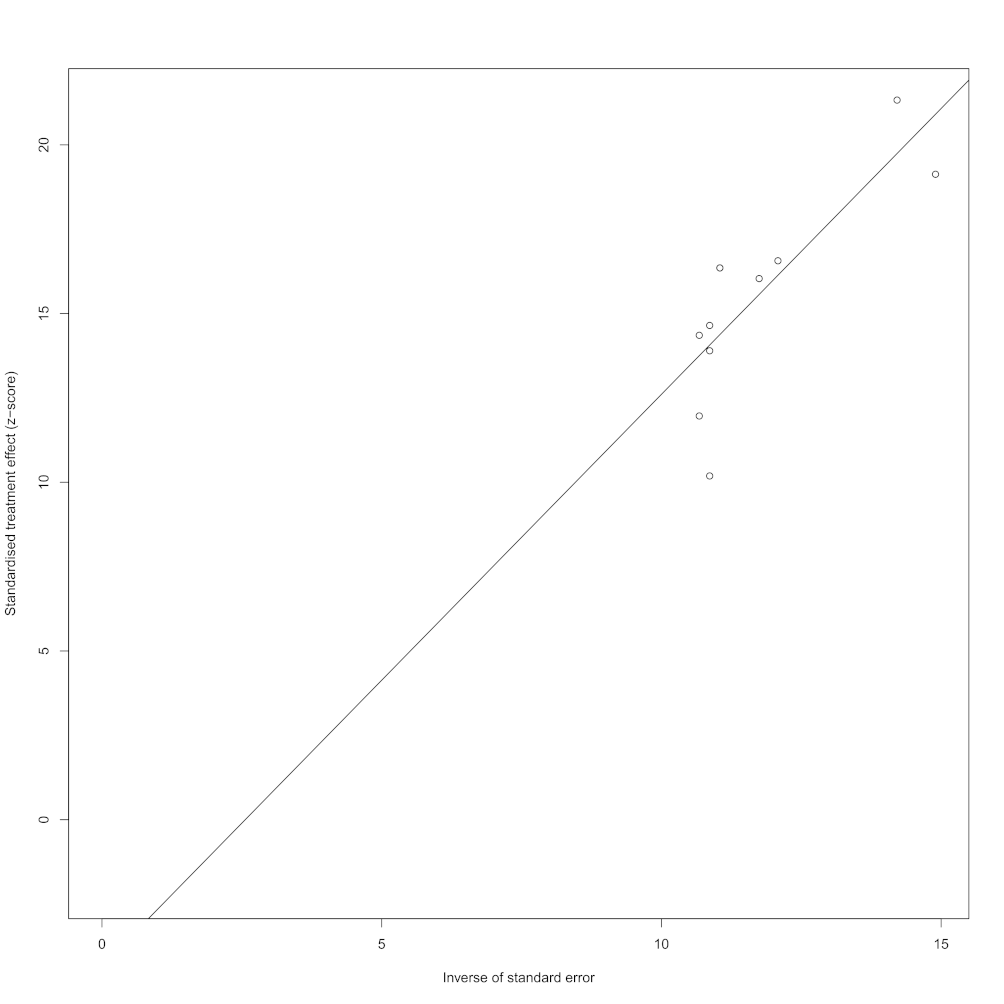


Figure 3 Egger test plot for R0

**Supplementary Figure 3.**Egger test plot for R_0_

**
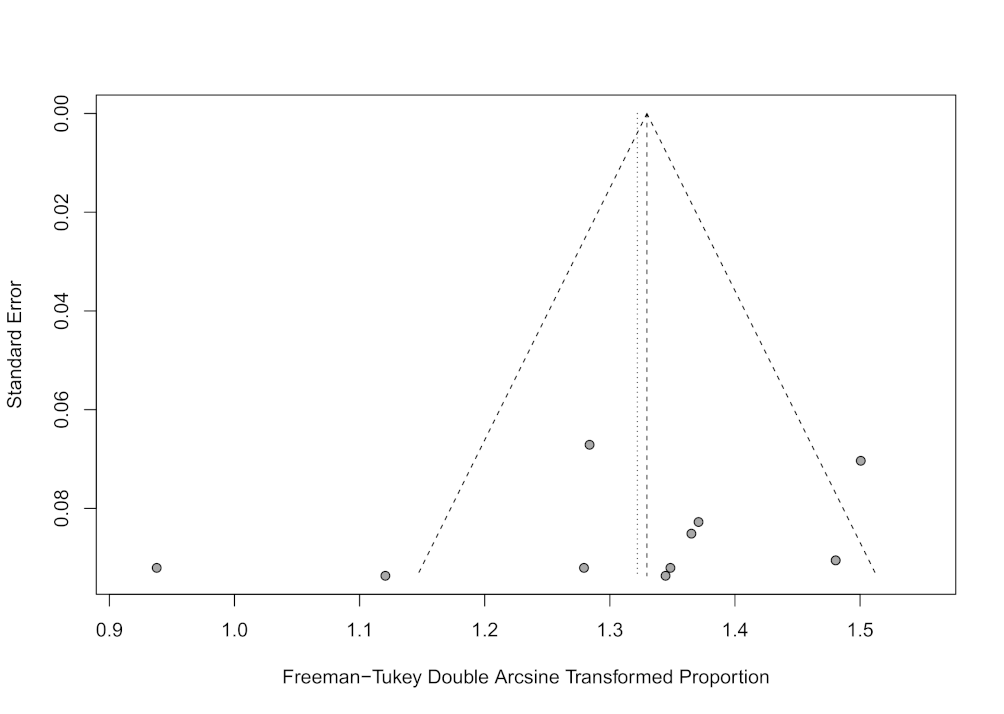
**

Figure 4 Funnel plot for R0

**Supplementary Figure 4.** Funnel plot for R_0_

_
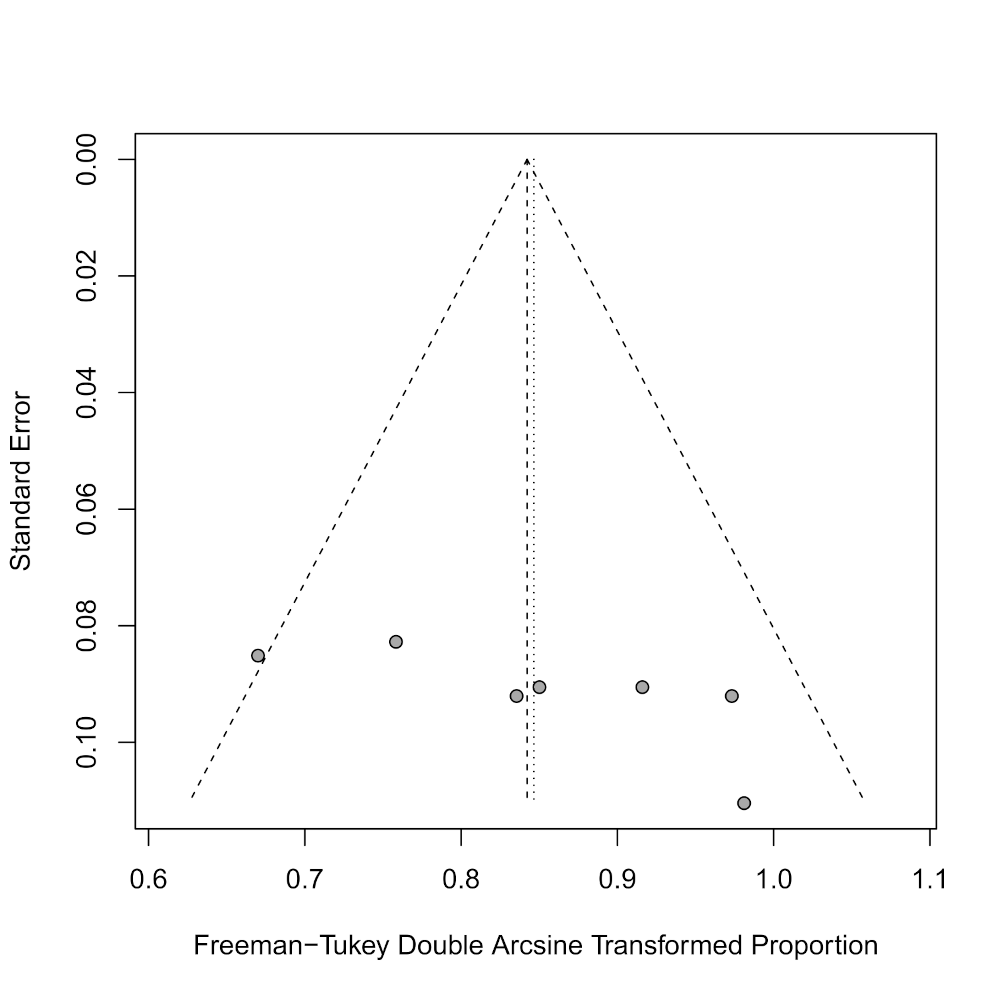
_

Figure 5 Funnel plot for MPR

**Supplementary Figure 5.**Funnel plot for MPR


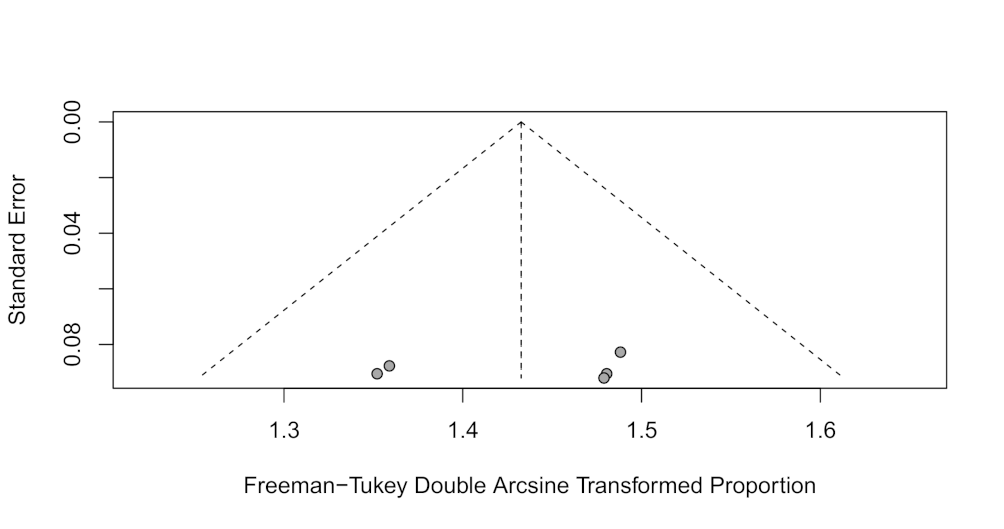


Figure 6 Funnel plot for DCR

**Supplementary Figure 6.**Funnel plot for DCR


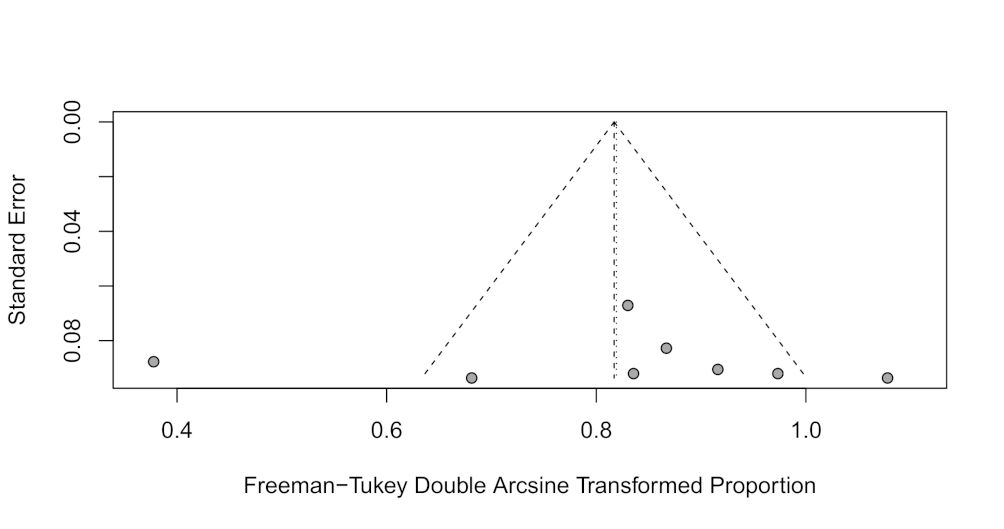


Figure 7 Funnel plot for ypN0 Rate

**Supplementary Figure 7.**Funnel plot for ypN0 Rate


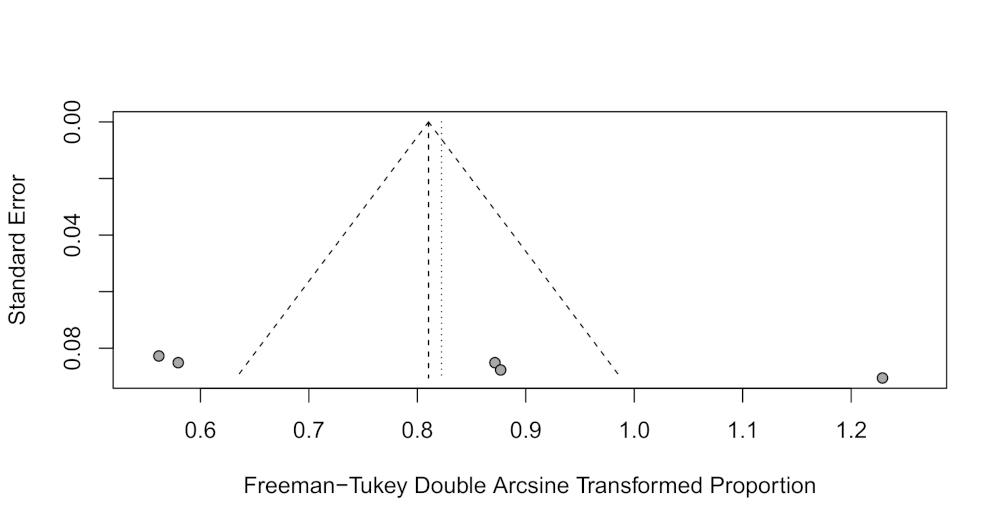


Figure 8 Funnel plot for trAEs

**Supplementary Figure 8.**Funnel plot for trAEs


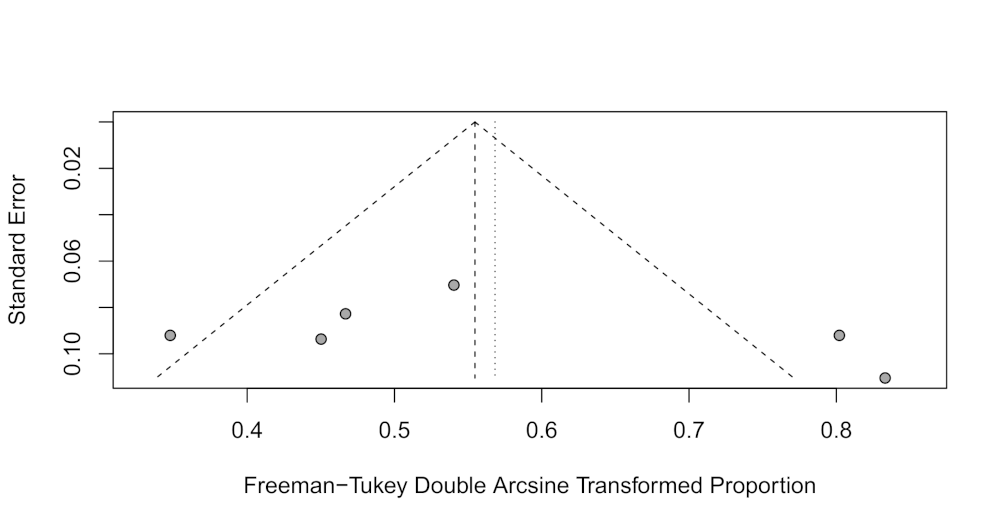


Figure 9 Funnel plot for Postoperative Complications

**Supplementary Figure 9.**Funnel plot for Postoperative Complications


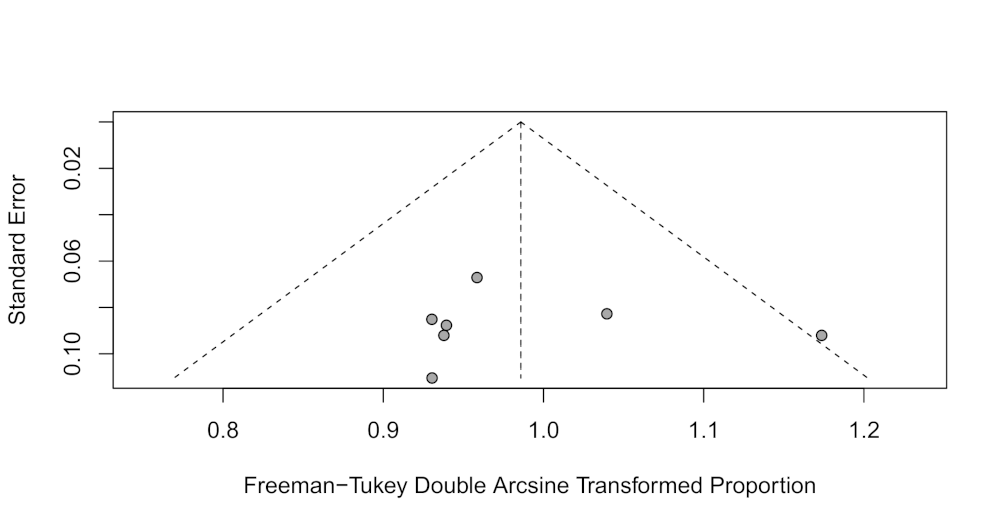


Figure 10 Tumor Downstaging

**Supplementary Figure 10.**Tumor Downstaging


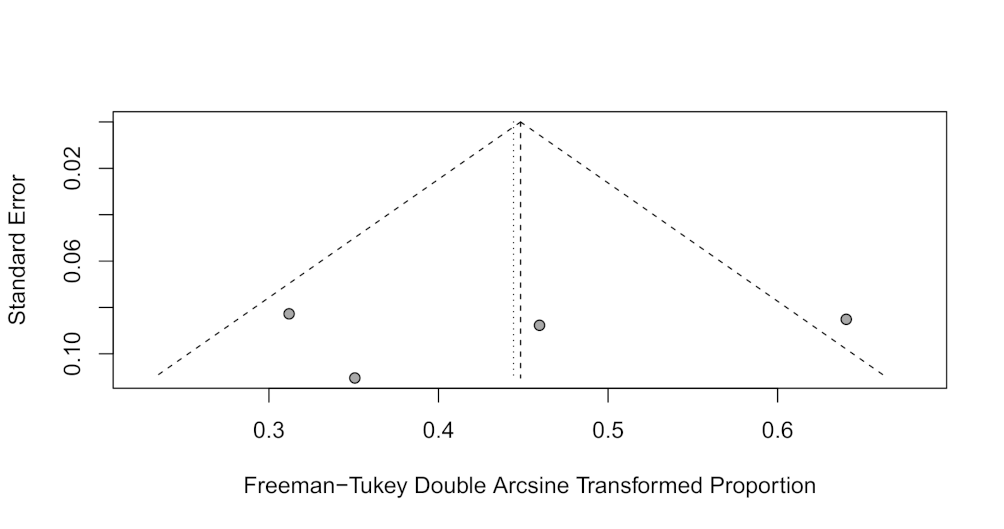


Figure 11 Funnel plot for irAEs

**Supplementary Figure 11.**Funnel plot for irAEs


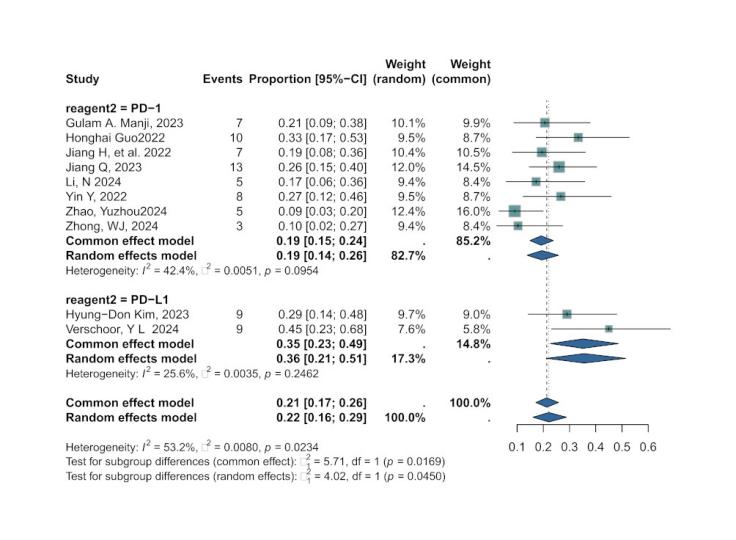


Figure 12

**Supplementary Figure 12.**Subgroup Analysis of Immune Checkpoint Inhibitors of pCR


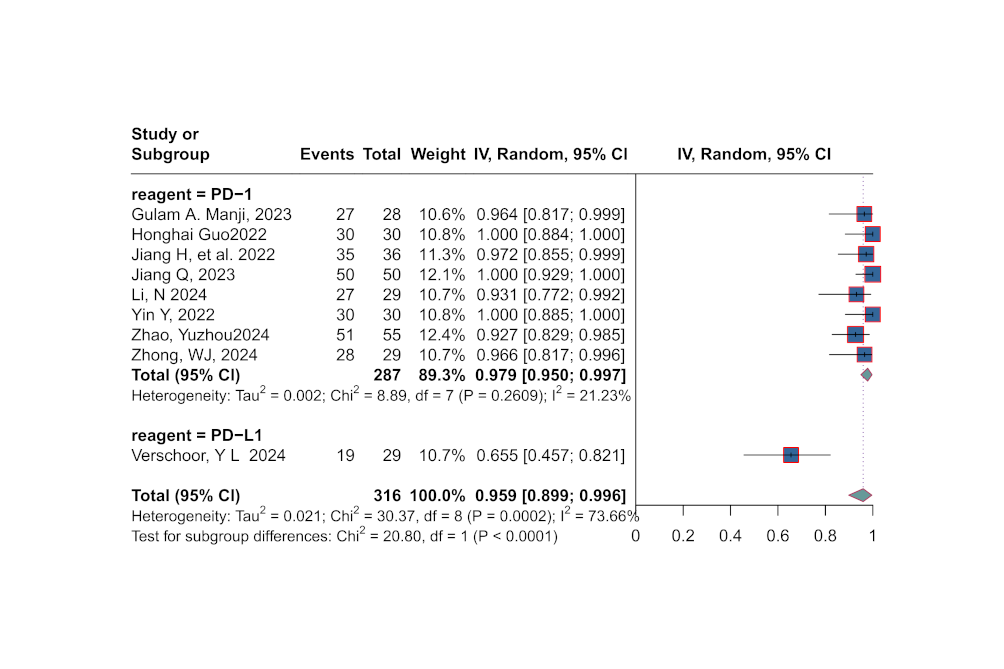


Figure 13

**Supplementary Figure 13.**Subgroup Analysis of Immune Checkpoint Inhibitors of R_0_.


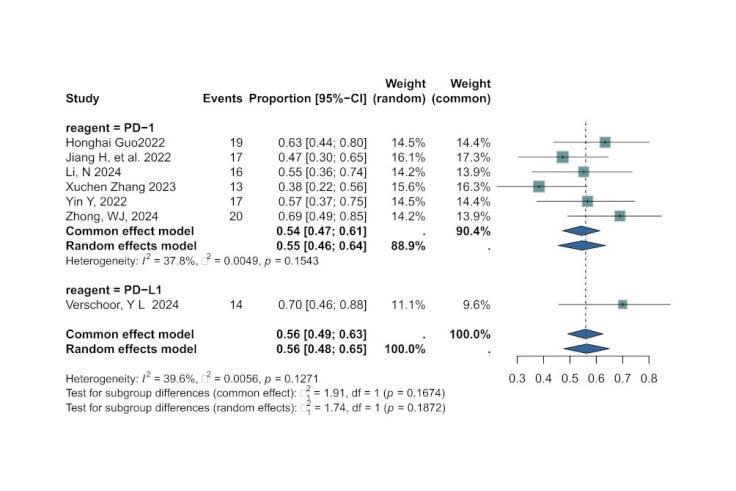


Figure 14

**Supplementary Figure 14.**Subgroup Analysis of Immune Checkpoint Inhibitors of MPR.


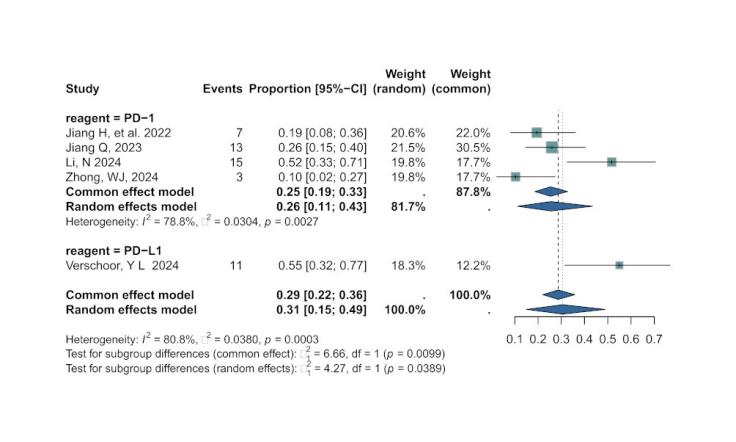


Figure 15

**Supplementary Figure 15.**Subgroup Analysis of Immune Checkpoint Inhibitors of Surgical Complications.


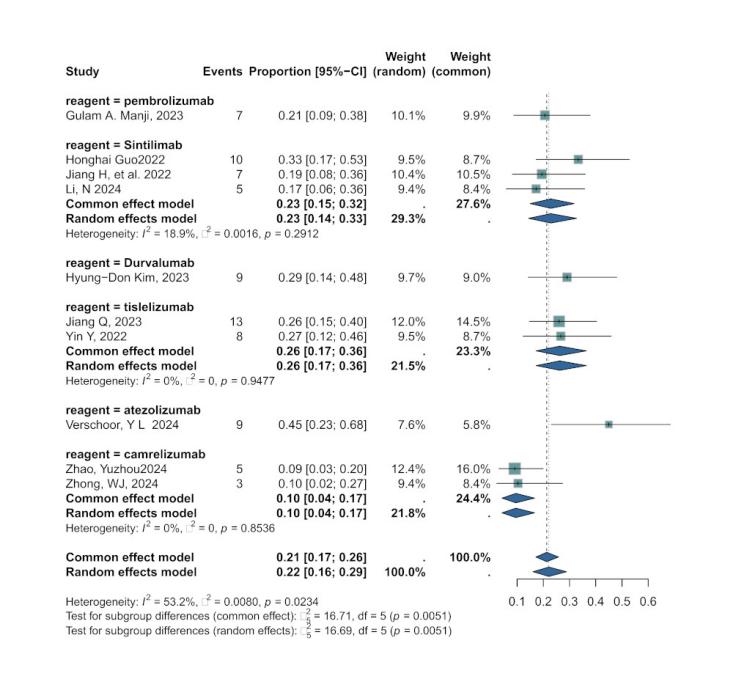


Figure 16

**Supplementary Figure 16.**Subgroup analysis of PD-1 inhibitor Species for pCR


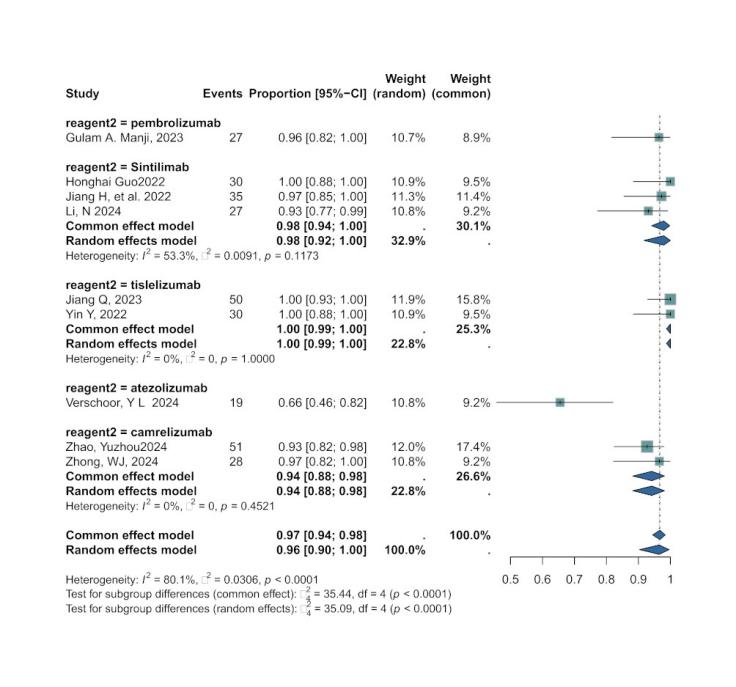


Figure 17

**Supplementary Figure 17.**Subgroup analysis of PD-1 inhibitor Species for R_0_

_
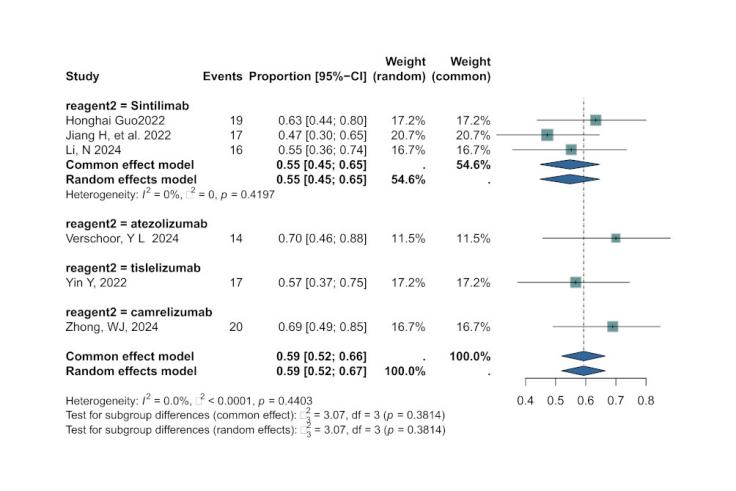
_

Figure 18

**Supplementary Figure 18.**Subgroup analysis of PD-1 inhibitor Species for MPR


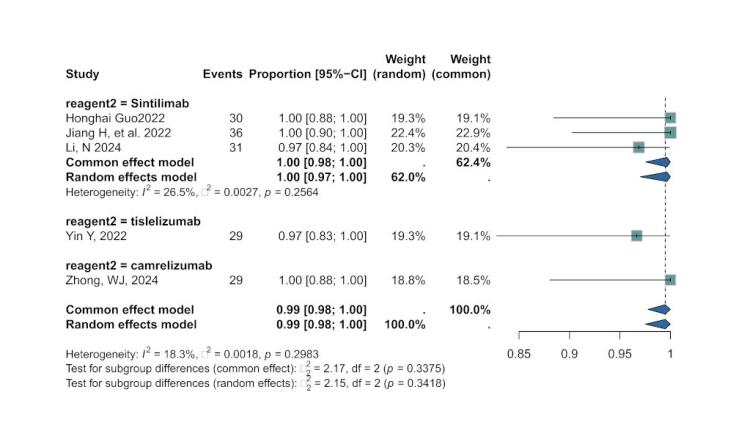


Figure 19

**Supplementary Figure 19.**Subgroup analysis of PD-1 inhibitor Species for DCR


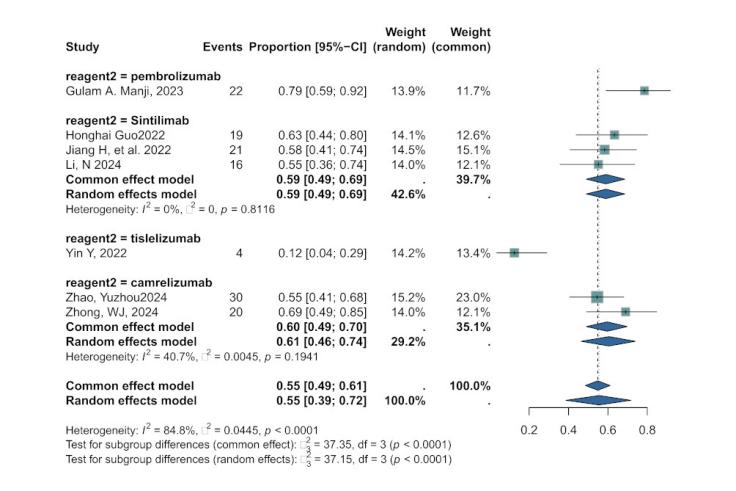


Figure 20

**Supplementary Figure 20.**Subgroup analysis of PD-1 inhibitor Species for ypN0 Rate


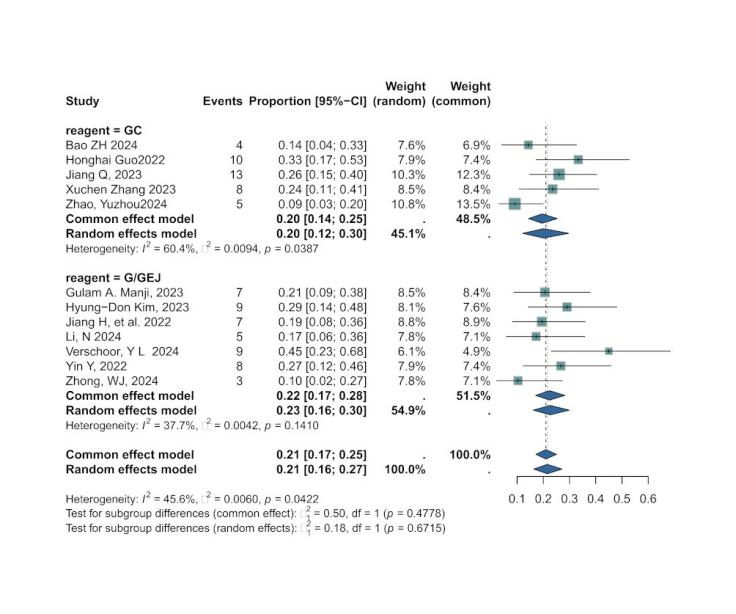


Figure 21

**Supplementary Figure 21.**Subgroup analysis of Anatomical Location for PCR Rate


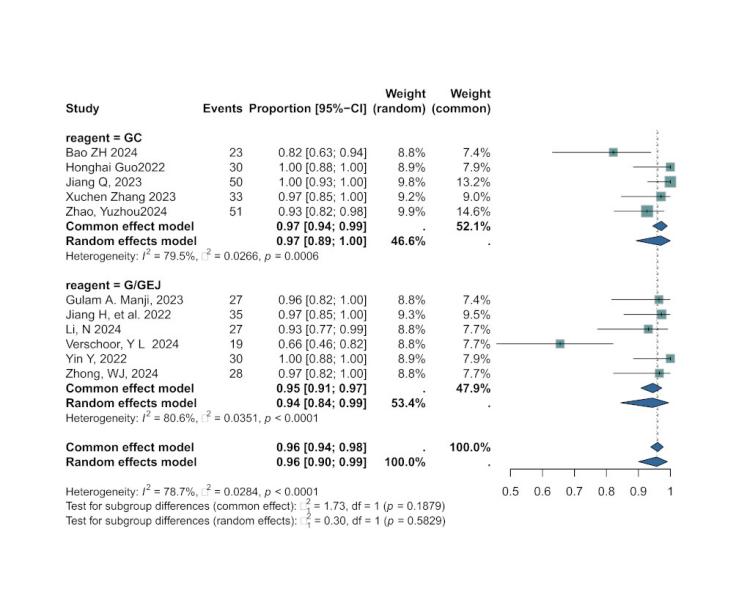


Figure 22

**Supplementary Figure 22.**Subgroup analysis of Anatomical Location for R0 Rate


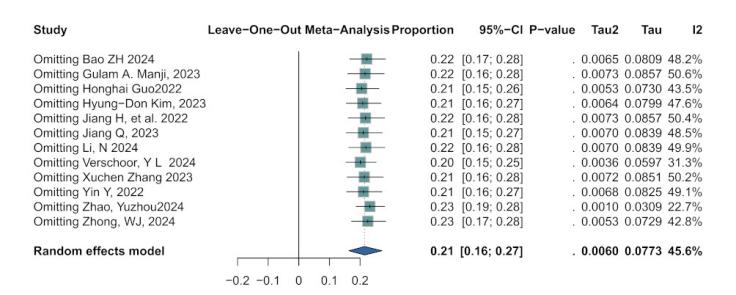


Figure 23

**Supplementary Figure 23.**Sensitivity Analysis for pCR


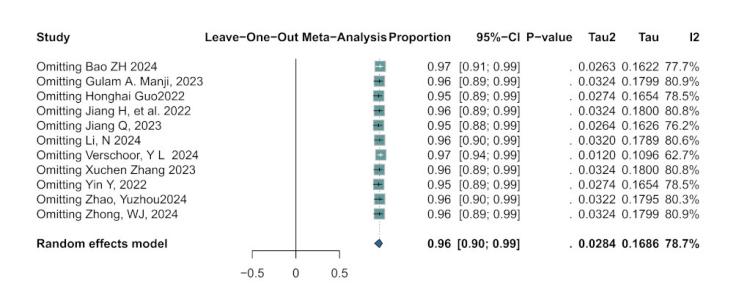


Figure 24

**Supplementary Figure 24.**Sensitivity Analysis for R0


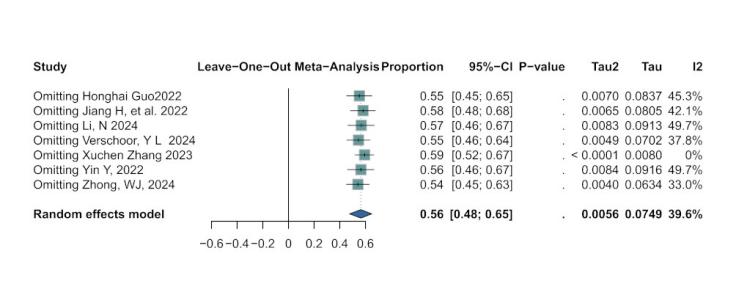


Figure 25

**Supplementary Figure 25.**Sensitivity Analysis for MPR


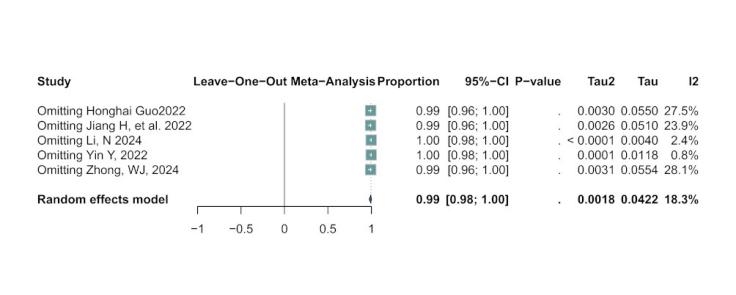


Figure 26

**Supplementary Figure 26.**Sensitivity Analysis for DCR


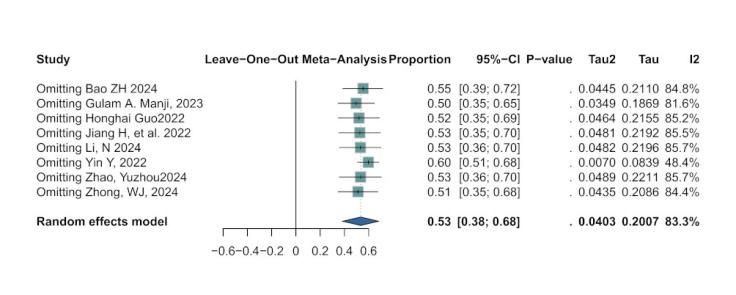


Figure 27

**Supplementary Figure 27.**Sensitivity Analysis for ypN0 Rate


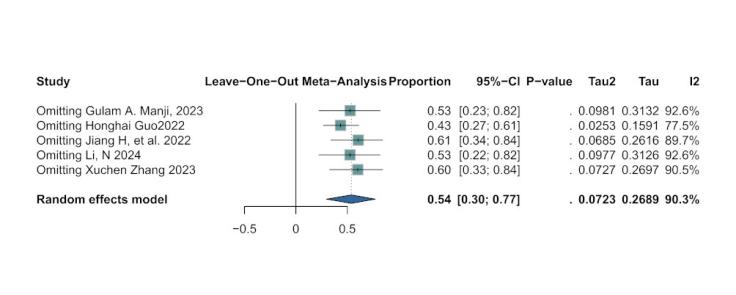


Figure 28

**Supplementary Figure 28.**Sensitivity Analysis for trAEs


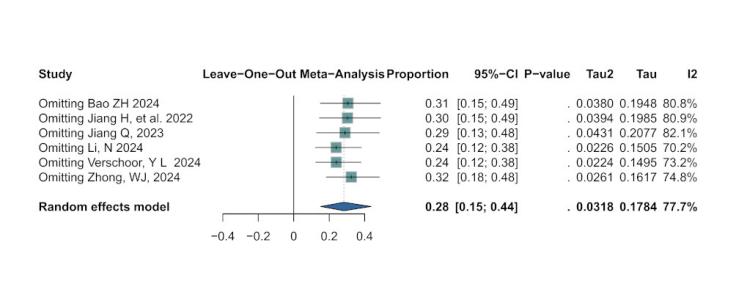


Figure 29

**Supplementary Figure 29.**Sensitivity Analysis for Postoperative Complications


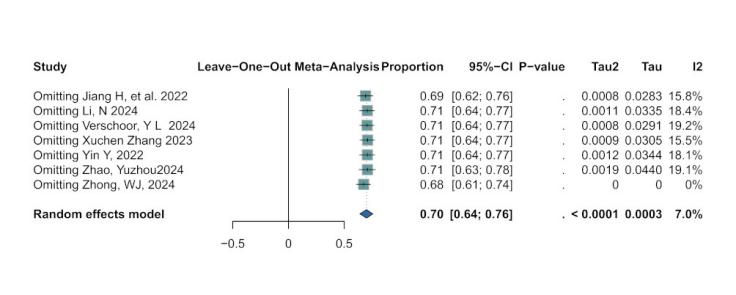


Figure 30

**Supplementary Figure 30.**Sensitivity Analysis for Tumor Downstaging


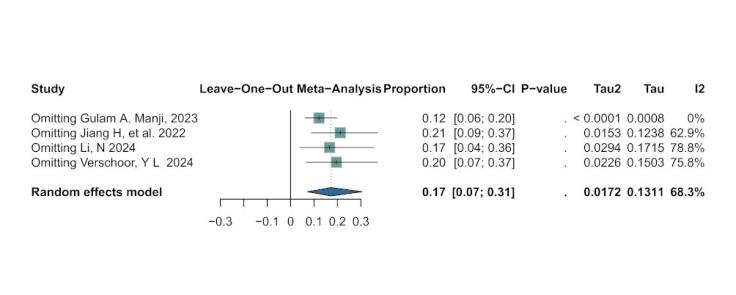


Figure 31

**Supplementary Figure 31.**Sensitivity Analysis for MPR irAEs


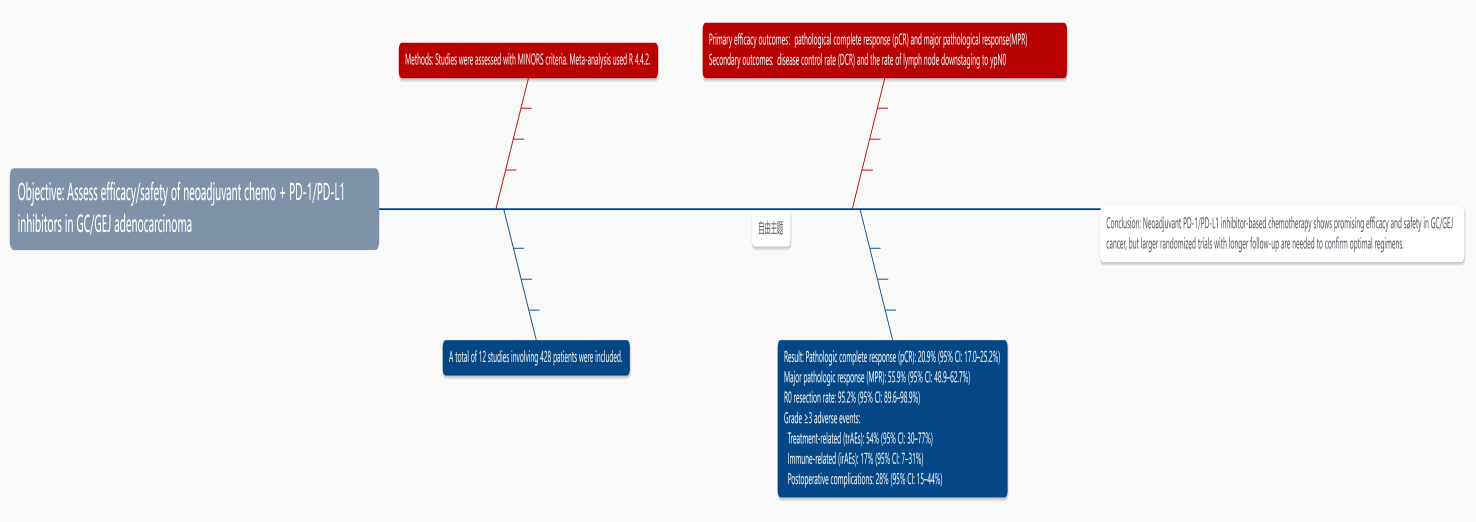


**Supplementary Figure 32:** graphical abstract

# 2.2 Supplementary Tables

# Supplementary Table1.

| **Authors** | **Q1** | **Q2** | **Q3** | **Q4** | **Q5** | **Q6** | **Q7** | **Q8** | **Q9-12** | **Score (quality)** |
| --- | --- | --- | --- | --- | --- | --- | --- | --- | --- | --- |
| Li, N 2024 | 2 | 2 | 2 | 2 | 2 | 2 | 2 | 1 | / | 15 |
| Yin, Y 2022 | 2 | 1 | 2 | 2 | 1 | 1 | 0 | 2 | / | 11 |
| Zhao, Yuzhou 2024 | 2 | 1 | 2 | 2 | 1 | 2 | 0 | 0 | / | 10 |
| Zhong, W J 2024 | 2 | 1 | 2 | 2 | 1 | 1 | 0 | 2 | / | 11 |
| Verschoor YL 2024 | 2 | 1 | 2 | 2 | 1 | 2 | 2 | 0 | / | 12 |
| Xuchen Zhang 2023 | 2 | 1 | 0 | 2 | 1 | 1 | 2 | 0 | / | 9 |
| Kim HD 2023 | 2 | 1 | 2 | 2 | 1 | 2 | 0 | 1 | / | 11 |
| Jiang H 2022 | 2 | 1 | 2 | 2 | 1 | 2 | 2 | 2 | / | 14 |
| Jiang Q 2023 | 2 | 1 | 1 | 2 | 1 | 1 | 2 | 1 | / | 11 |
| Bao, 2024 | 2 | 2 | 0 | 2 | 2 | 1 | 0 | 0 | / | 9 |
| Guo, HH 2022 | 2 | 1 | 2 | 2 | 1 | 2 | 1 | 0 | / | 11 |
| Manji, GA 2023 | 2 | 1 | 0 | 2 | 2 | 1 | 0 | 0 | / | 8 |

**eTable 1：Assessment of the quality of included studies according to MINORS.**

Quality of non-comparative studies was determined based on the first eight items; the last four items were only used to assess comparative studies.

Checklist items: 1, a stated aim of the study; 2, inclusion of consecutive patients; 3, prospective collection of data; 4, endpoints appropriate to study aim; 5, unbiased assessment of study endpoint; 6, follow-up period appropriate to the major endpoint; 7, <5% lost to follow-up; 8, adequate control group; 9, contemporary groups; 10, baseline equivalence of groups; 11, prospective calculation of study size; 12, adequate statistical analyses. Items are scored as 0 (not reported); 1 (reported but inadequate); or 2 (reported and adequate). The maximum possible score is 24 points.For non-comparative studies, an overall score > 12 = high; 8–12 = intermediate; < 8 = low. For comparative studies, > 18 = high; 12–18 = intermediate; < 12 = low.
